# Genetic analyses of human fetal retinal pigment epithelium gene expression suggest ocular disease mechanisms

**DOI:** 10.1038/s42003-019-0430-6

**Published:** 2019-05-20

**Authors:** Boxiang Liu, Melissa A. Calton, Nathan S. Abell, Gillie Benchorin, Michael J. Gloudemans, Ming Chen, Jane Hu, Xin Li, Brunilda Balliu, Dean Bok, Stephen B. Montgomery, Douglas Vollrath

**Affiliations:** 1 Department of Biology, Stanford University, Stanford, CA 94305 USA; 2 Department of Genetics, Stanford University School of Medicine, Stanford, CA 94305 USA; 3 Program in Biomedical Informatics, Stanford University School of Medicine, Stanford, 94305 CA USA; 4 Department of Ophthalmology, Jules Stein Eye Institute, UCLA, Los Angeles, 90095 CA USA; 5 Department of Pathology, Stanford University School of Medicine, Stanford, CA 94305 USA

**Keywords:** Gene expression, Computational biology and bioinformatics, Macular degeneration

## Abstract

The retinal pigment epithelium (RPE) serves vital roles in ocular development and retinal homeostasis but has limited representation in large-scale functional genomics datasets. Understanding how common human genetic variants affect RPE gene expression could elucidate the sources of phenotypic variability in selected monogenic ocular diseases and pinpoint causal genes at genome-wide association study (GWAS) loci. We interrogated the genetics of gene expression of cultured human fetal RPE (fRPE) cells under two metabolic conditions and discovered hundreds of shared or condition-specific expression or splice quantitative trait loci (e/sQTLs). Co-localizations of fRPE e/sQTLs with age-related macular degeneration (AMD) and myopia GWAS data suggest new candidate genes, and mechanisms by which a common *RDH5* allele contributes to both increased AMD risk and decreased myopia risk. Our study highlights the unique transcriptomic characteristics of fRPE and provides a resource to connect e/sQTLs in a critical ocular cell type to monogenic and complex eye disorders.

## Abstract (web summary)

Boxiang Liu, Melissa Calton et al. report expression and splice quantitative trait locus analyses for human fetal retinal epithelium (fRPE) cells under two metabolic conditions. They identify loci linked to inherited ocular diseases and new candidate genes for age-related macular degeneration and myopia.

## Introduction

The importance of vision to humans and the accessibility of the eye to examination have motivated the characterization of more than one thousand genetic conditions involving ocular phenotypes^1^. Among these, numerous monogenic diseases exhibit considerable inter-familial and intra-familial phenotypic variability^2–7^. Imbalance in allelic expression of a handful of causative genes has been documented^8^, but few common genetic variants responsible for such effects have been discovered.

Complementing our knowledge of numerous monogenic ocular disorders, recent genome-wide association studies (GWAS)^9^ have identified hundreds of independent loci associated with polygenic ocular phenotypes such as age-related macular degeneration (AMD), the leading cause of blindness in elderly individuals in developed countries^10,11^, and myopia, the most common type of refractive error worldwide and an increasingly common cause of blindness^12–14^. Despite the rapid success of GWAS in mapping novel ocular disease susceptibility loci, the functional mechanisms underlying these associations are often obscure.

Connecting changes in molecular functions such as gene expression and splicing with specific GWAS genomic variants has aided the elucidation of functional mechanisms. Non-coding variants account for a preponderance of the most significant GWAS loci^15,16^, and most expression quantitative trait loci (eQTLs) map to non-coding variants^17^. Thousands of eQTLs have been found in a variety of human tissues^18^, but ocular cell-types are underrepresented among eQTL maps across diverse tissues.

The retinal pigment epithelium (RPE) is critical for eye development^19^ and for an array of homeostatic functions essential for photoreceptors^20^. Variants of RPE-expressed genes have been associated with both monogenic and polygenic ocular phenotypes, including AMD and myopia. We recently implicated an eQTL associated with an RPE-expressed gene as modulating the severity of inherited photoreceptor degeneration in mice^21^.

To investigate the potential effects of genetically encoded common variation on human RPE gene expression, we set out to identify eQTLs and splice quantitative trait loci (sQTLs) for human fetal RPE (fRPE) cells cultured under two metabolic conditions. Here we describe hundreds of loci of each type, some of which are condition-specific, and connect the mitochondrial oxidation of glutamine with increased expression of lipid synthesis genes, a pathway important in AMD. We find that common variants near genes with disproportionately high fRPE expression explain a larger fraction of risk for both AMD and myopia than variants near genes enriched in non-ocular tissues. We show that a particular variant in *RDH5* is associated with increased skipping of a coding exon, nonsense-mediated decay (NMD) of the aberrant transcript, and three-fold lower minor allele-specific expression. The e/sQTL marked by this variant colocalizes with high statistical significance with GWAS loci for both AMD and myopia risk, but with opposing directions of effect. Our study lays a foundation for linking e/sQTLs in a critical ocular cell type to mechanisms underlying monogenic and polygenic eye diseases.

## Results

### The transcriptome of human fRPE cells

We studied 23 primary human fRPE lines (Supplementary Data 1), all generated by the same method in a single laboratory^22^ and cultured for at least 10 weeks under conditions that promote a differentiated phenotype^23^. DNA from each line was genotyped at 2,372,784 variants. Additional variants were imputed and phased using Beagle v4.1^24^ against 1000 Genomes Phase 3^25^ for a total of ~13 million variants after filtering and quality control (see Methods section). Comparison of fRPE chromosome 1 genotypes to those of 104 samples from 1000 Genomes indicated that our cohort is mostly African American in origin, with 4 samples of European ancestry (Supplementary Fig. 1).

**Fig. 1:**
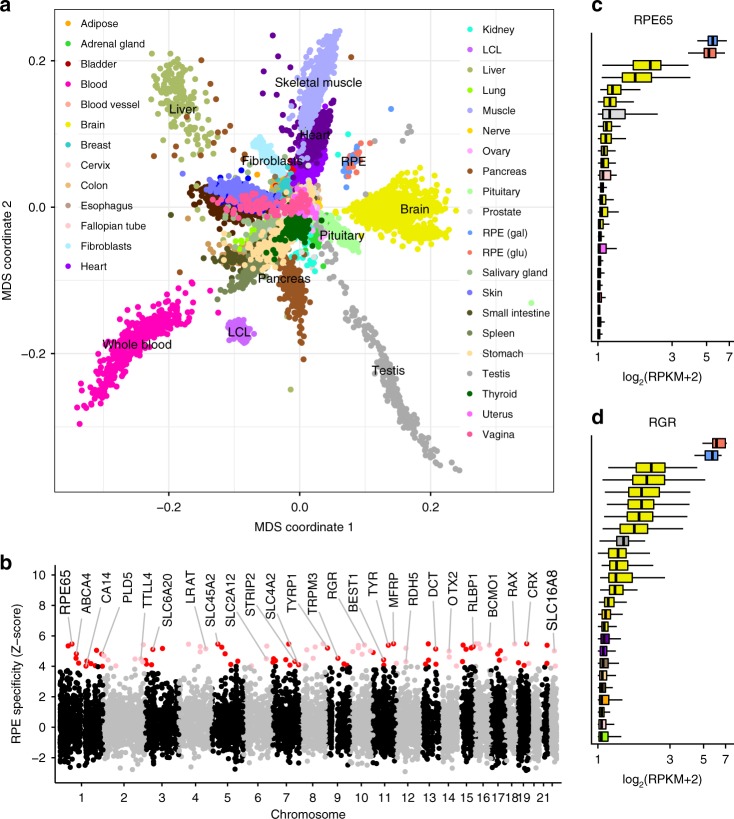
Characteristics of the fRPE transcriptome. **a** Multidimensional scaling against GTEx tissues locates fRPE near heart, skeletal muscle, and brain samples. **b** A subset of the fRPE-selective gene set defined by *z*-score >4 is shown including RPE signature genes such as *RPE65* and new genes such as *TYR*. Red/pink dots indicate fRPE-selective genes with *z*-score >4 in both glucose and galactose conditions. **c**, **d** Two examples of the expression levels of fRPE-selective genes in various GTEx tissues. Only the top 25 tissues are plotted for visual clarity. For **a**, **c**, and **d**, red indicates fRPE glucose condition and blue indicates fRPE galactose condition. For **c** and **d**, each element of the boxplot is defined as follows: centerline, median; box limits, upper and lower quartiles; whiskers, 1.5× interquartile range

Our goal was to identify RPE eQTLs relevant to the tissue’s role in both developmental and chronic eye diseases. The balance between glycolytic and oxidative cellular energy metabolism changes during development and differentiation^26^, and loss of RPE mitochondrial oxidative phosphorylation capacity may contribute to the pathogenesis of AMD^27^, among other mechanisms. We therefore obtained transcriptional profiles of each fRPE line cultured in medium that favors glycolysis (glucose plus glutamine) and in medium that promotes oxidative phosphorylation (galactose plus glutamine)^28^. We performed 75-base paired-end sequencing to a median depth of 52.7 million reads (interquartile range: 45.5 to 60.1 million reads) using a paired sample design to minimize batch effects in differential expression analysis (Supplementary Data 2). To determine the relationship between primary fRPE and other tissues, we visualized fRPE in the context of 53 tissues from the GTEx Project v7^18^. The fRPE samples formed a distinct cluster situated between heart and skeletal muscle and brain (Fig. 1a), tissues that, like the RPE, are metabolically active and capable of robust oxidative phosphorylation.

To identify genes with disproportionately high levels of expression in the fRPE, we compared the median reads per kilobase of transcript per million mapped reads (RPKMs) of fRPE genes against GTEx tissues. We defined fRPE-selective genes as those with median expression at least four standard deviations above the mean (see Methods section). Under this definition, we found 100 protein-coding genes and 30 long non-coding RNAs (lncRNAs) to be fRPE-selective (Fig. 1b and Supplementary Data 3). Multiple previously defined RPE “signature” genes^29–31^ are present in our list including *RPE65* (Fig. 1c) and *RGR* (Fig. 1d). Using this set of genes, we performed Gene Set Enrichment Analysis (GSEA)^32^ against 5,917 gene ontology (GO) annotations^33^. The two gene sets most enriched with fRPE-selective genes were *pigment granule* and *sensory perception of light stimulus* (FDR < 1 × 10^−3^), consistent with the capacity of fRPE to produce melanin and the tissue’s essential role in the visual cycle. Supplementary Data 4 lists the 29 GO pathways enriched using a conservative FWER < 0.05. Recurrent terms in enriched pathway annotations such as pigmentation, light, vitamin, protein translation, endoplasmic reticulum and cellular energy metabolism suggest specific functions that are central to fRPE and outer retinal homeostasis.

### Transcriptomic differences across two metabolic conditions

**Fig. 2:**
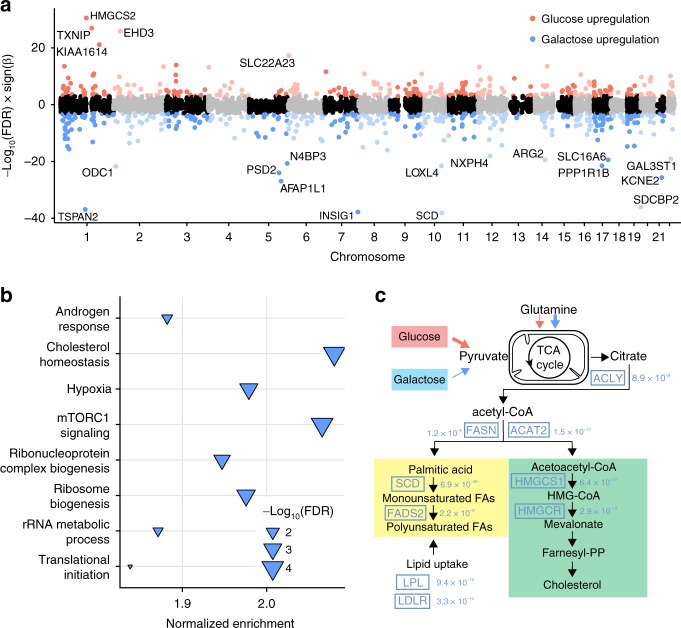
Differential expression across two metabolic conditions. **a** Transcriptome-wide differential expression patterns: red indicates upregulated in glucose, blue indicates upregulated in galactose. **b** Gene set enrichment analysis of differentially expressed genes. The pathway most enriched is cholesterol homeostasis (upregulated in galactose condition). **c** Key genes involved in cholesterol biosynthesis and import are upregulated in response to the increased oxidation of glutamine that occurs in the galactose condition. Estimated FDR values are shown next to the gene names

To gain insight into the response of fRPE cells to altered energy metabolism, we compared gene expression between the two culture conditions using DESeq2^34^, correcting for sex, ancestry, RIN, and batch (see Methods section). A total of 837 protein coding and lncRNA genes showed evidence of significant differential expression (FDR < 1 × 10^−3^, Fig. 2a and Supplementary Data 5). Notably, three of the top ten differentially expressed genes are involved in lipid metabolism (*SCD*, *INSIG1*, and *HMGCS2* in order). *SCD* codes for a key enzyme in fatty acid metabolism^35^, and its expression in RPE is regulated by retinoic acid^36^. *INSIG1* encodes an insulin-induced protein that regulates cellular cholesterol concentration^37^. *HMGCS2* encodes a mitochondrial enzyme that catalyzes the first step of ketogenesis^38^, and this enzyme plays a crucial role in phagocytosis-dependent ketogenesis in fRPE^39^. To understand the broader impact induced by changes in energy metabolism, we performed pathway enrichment analysis using GSEA^32^ and found that the top two upregulated pathways in galactose medium are cholesterol homeostasis and mTORC1 signaling (FDR < 1 × 10^−4^, Fig. 2b). Consistent with the cholesterol finding, forcing cells to rely primarily on oxidation of glutamine for ATP generation increases expression of a suite of genes that promotes lipid synthesis and import (Fig. 2c).

### fRPE-selective genes are enriched in genetic ocular diseases

**Fig. 3:**
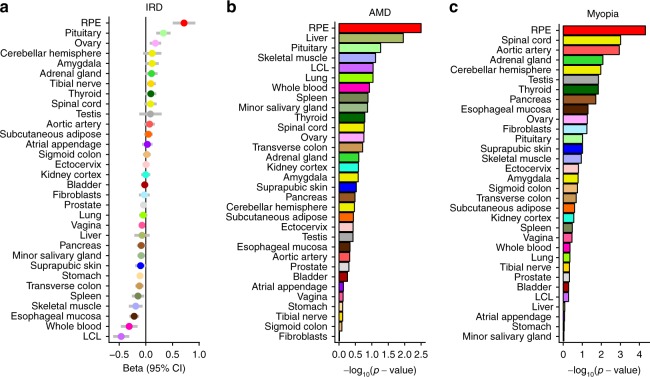
fRPE-selective genes are enriched in monogenic and polygenic diseases. **a** Genes causal for inherited retinal disorders (IRD) have elevated expression in fRPE. **b**, **c** Variants near RPE-selective genes explain a larger proportion of AMD (**b**) and myopia (**c**) risk than those near GTEx tissue-selective genes. The red bar represents the top 500 fRPE-selective genes

Disease-associated genes can have elevated expression levels in effector tissues^40^. To determine whether ocular disease genes have elevated expression levels in fRPE, we used a manually curated list of 257 ocular disease-related genes^41^ (see Methods section). Compared to all other protein-coding genes, ocular disease-related genes are more specific to fRPE (two-sided *t*-test *p*-value: 1.6 × 10^−10^). Further, ocular disease gene expression demonstrated a higher specificity to fRPE than to GTEx tissues (Fig. 3a), suggesting fRPE as a model system for a number of eye diseases. As a control, we repeated the analysis for epilepsy genes (*n* = 189) and observed elevated expression levels in brain tissues as we expected (Supplementary Fig. 2).

Unlike Mendelian ocular diseases, polygenic ocular disorders are characterized by variants with smaller effect sizes scattered throughout the genome. Using two well-powered GWAS of AMD^42^ and myopia^43^, we performed stratified linkage disequilibrium (LD) score regression to determine the heritability explained by fRPE. Using a previously established pipeline^44^, we selected the top 500 tissue-enriched genes for fRPE and various GTEx tissues and assigned variants within one kilobase of these genes to each tissue (see Methods section). Risk variants for both AMD and myopia were more enriched around fRPE-selective genes than GTEx tissue-selective genes (Fig. 3b, c). As an assessment of the robustness of the LD score regression results, we repeated the analysis with the top 200 and 1000 tissue-specific genes. A high ranking for fRPE was consistent across all three cutoffs (Supplementary Fig. 3).

### e/sQTL discovery

**Fig. 4:**
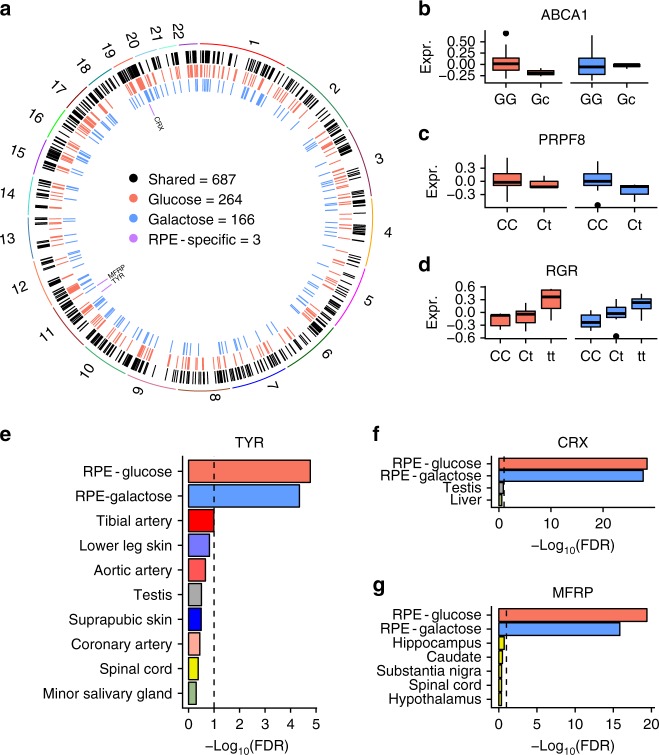
Landscape of genetic regulation of RPE gene expression. **a** We discovered 687, 264, and 166 eQTLs that are shared, glucose-specific, and galactose-specific, respectively. Comparison with GTEx eGenes revealed three shared eGenes that are currently unique to fRPE. **b** A glucose-specific eQTL in *ABCA1*. **c** A galactose-specific eQTL in *PRPF8*. **d** A shared eQTL in *RGR*. The *y*-axis of panels **b**–**d** denotes normalized expression values. **e**–**g** Evidence for fRPE-specificity for three eQTLs compared to GTEx. Black dashed lines indicate FDR = 0.1. Minor alleles are indicated by lowercase. For **b**, **c**, and **d**, each element of the boxplot is defined as follows: centerline, median; box limits, upper and lower quartiles; whiskers, 1.5× interquartile range

To determine the genetic effects on gene expression in fRPE, we used RASQUAL^45^ to map eQTLs by leveraging both gene-level and allele-specific count information to boost discovery power. Multiple-hypothesis testing for both glucose and galactose conditions was conducted jointly with a hierarchical procedure called TreeQTL^46^. At FDR < 0.05, we found 687 shared, 264 glucose-specific, and 166 galactose-specific eQTLs (Table 1, Supplementary Data 6 and 7, Fig. 4a and Supplementary Figs. 4 and 5). An example of a shared eQTL is *RGR* (Fig. 4d), which encodes a G protein-coupled receptor that is mutated in retinitis pigmentosa^47^. An example of a glucose-specific eQTL is *ABCA1* (Fig. 4b), which encodes an ATP-binding cassette transporter that regulates cellular cholesterol efflux^48^. Common variants near *ABCA1* have been associated with glaucoma^49^ and AMD^42^. An example of a galactose-specific eQTL is *PRPF8* (Fig. 4c), which encodes a splicing factor^50^. *PRPF8* mutations are a cause of autosomal dominant retinitis pigmentosa^51^ and lead to RPE dysfunction in a mouse model^52^.

**Table 1:** Expression QTL discoveries

Trait type	No. tested	No. of eQTLs (FDR < 0.05)		
		Glucose	Galactose	Shared
Protein coding	12,515	254	163	656
lincRNA	582	10	3	31
Total	13,097	264	166	687

Differential expression alone is unlikely to account for the condition-specific nature of the eQTLs we identified because only about a quarter are differentially expressed (FDR < 0.05) and almost all of these exhibit an absolute fold change of less than two. Rather, it is likely that regulatory specificity is the underlying cause of these eQTLs. We therefore used HOMER^53^ to identify transcription factor binding motifs enriched around metabolic-specific eQTLs (see Methods section). Two motifs, TEAD1 (*p* < 1 × 10^−6^) and ZEB1 (*p* < 1 × 10^−3^), are among the top five motifs in the galactose condition (Supplementary Data 8). TEAD1 is known to play a role in aerobic glycolysis reprogramming^54^, and ZEB1 is known to render cells resistant to glucose deprivation^55^. We did not find enriched motifs in the glucose condition for transcription factors with well-known metabolic functions.

We compared fRPE to GTEx eGenes using a previously established two-step FDR approach^56^. We used fRPE-shared eGenes (FDR < 0.05 in both metabolic conditions) as the discovery set to remove any treatment-dependent regulatory effect, and used GTEx eGenes with a relaxed threshold (FDR < 0.1) as the replication set. eGenes from the discovery set not recapitulated in the replicated set were defined as fRPE-selective eGenes. This approach returned three genes (Fig. 4e–g): *TYR*, encoding an oxidase controlling the production of melanin; *CRX*, encoding a transcription factor critical for photoreceptor differentiation; and *MFRP*, encoding a secreted WNT ligand important for eye development. The *TYR* eQTL maps to a variant (rs4547091) previously described as located in an OTX2 binding site and responsible for modulating *TYR* promoter activity in cultured RPE cells^57^. All three genes are also fRPE-selective genes (Fig. 1), suggesting that apparent regulatory specificity is the by-product of expression selectivity. We also compared our eGenes to the EyeGEx database^58^. Among the 687 eGenes shared across both conditions, 498 (72.5%) are also eGenes reported in EyeGEx.

We also assessed the genetic effect on splicing by quantifying intron usages with LeafCutter^59^ and mapping splicing quantitative trait loci (sQTL) with FastQTL^60^ in permutation mode to obtain intron-level *p*-values. Following an established approach^59^, we used a conservative Bonferroni correction across introns within each intron cluster and calculated FDR across cluster-level *p*-values (see Methods section). We found 210 and 193 sQTLs at FDR < 0.05 for glucose and galactose conditions, respectively (Table 2, Supplementary Data 9 and 10). The top sQTL in the glucose condition regulates splicing in *ALDH3A2* (FDR < 2.06 × 10^−9^), which codes for an aldehyde dehydrogenase isozyme involved in lipid metabolism^61^. Mutations in this gene cause Sjogren-Larsson syndrome^62^, which can affect the macular RPE^63^. The top sQTL in the galactose condition regulates splicing of transcripts encoding *CAST*, a calcium-dependent protease inhibitor involved in the turnover of amyloid precursor protein^64^.

**Table 2:** Splicing QTL discoveries

Condition	Trait type	No. tested	No. of sQTLs		
			FDR < 0.05	FDR < 0.01	FDR < 0.001
Glucose	Protein coding	15,516	205	129	66
	lncRNA	187	5	4	4
	Total	15,703	210	133	70
Galactose	Protein coding	16,239	188	112	56
	lncRNA	196	5	4	2
	Total	16,435	193	116	58

### Fine mapping of complex ocular disease risk loci

**Fig. 5:**
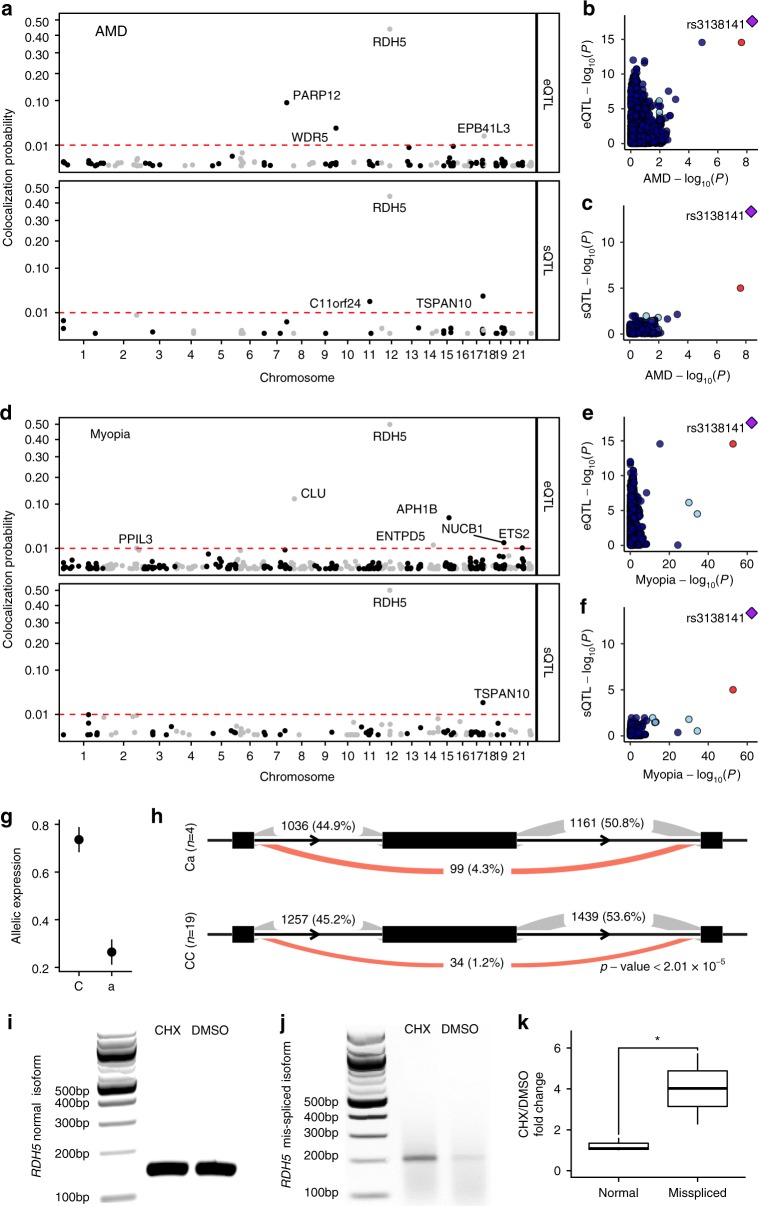
Fine mapping of disease-associated variants using fRPE gene regulation. **a** Colocalization posterior probability for fRPE e/sQTLs with AMD. **b**, **c** Scatter plots demonstrate clear colocalization between AMD GWAS signal at rs3138141 and *RDH5* eQTL (**b**) and sQTL (**c**). **d** Colocalization posterior probability for fRPE e/sQTLs with myopia. **e**, **f** Scatter plots demonstrate clear colocalization between myopia GWAS signal at rs3138141, the same variant identified for AMD, and *RDH5* eQTL (**e**) and sQTL (**f**). **a**–**f** Colocalization results are with glucose QTLs. Galactose QTL colocalizations can be found in Figs. S18–19. **g** Relative allelic expression estimated by RASQUAL with 95% confidence intervals is shown. **h** Increased skipping of *RDH5* exon 3 (middle black rectangle) is associated with the minor allele at rs3138141. The average read counts are shown for three splice junctions in groups of fRPE cells with different genotypes. The proportion of counts for all three sites for a given junction and genotype is shown in parenthesis. Exon and intron lengths are not drawn to scale. Minor alleles are indicated by lowercase. **i** Gel image showing *RHD5* normal isoform amplified from CHX or DMSO treated ARPE-19 cells. **j** Gel image showing *RHD5* mis-spliced isoform amplified from CHX or DMSO treated ARPE-19 cells. **k** Relative fold change between CHX and DMSO treatments for normal and mis-spliced RNA isoforms. Error bars indicate standard error of the mean for *n* = 3 independent experiments. *p < 0.05

To assess whether specific instances of GWAS signals can be explained by eQTL or sQTL signals, we performed colocalization analysis with a modified version of eCAVIAR^65^ (see Methods section). All variants within a 500-kilobase window around any GWAS (*p*-value < 1 × 10^−4^) or QTL (*p*-value < 1 × 10^−5^) signal were used as input to eCAVIAR, and any locus with colocalization posterior probability (CLPP) >0.01 was considered significant. To identify condition-specific colocalization events, we ran eCAVIAR separately for two metabolic conditions (see Methods section). For the AMD GWAS, we identified four eQTL colocalization events for each condition (Supplementary Fig. 6). One of these, *WDR5*, demonstrates glucose-specific colocalization (CLPP: glucose = 0.033 and galactose = 0.002). For the myopia GWAS, we identified three and seven colocalization events for galactose and glucose conditions, respectively. Three are condition specific (*PDE3A*, *ETS2*, and *ENTPD5*; Supplementary Fig. 7). For example, *PDE3A*, shows galactose-specific colocalization (CLPP: glucose = 0.0004; galactose = 0.014). eQTLs at *PARP12* and *CLU* colocalized with AMD and myopia signals, respectively, under both conditions (Fig. 5a, d, Supplementary Figs. 6 and 7). While neither locus reached genome-wide significance in the respective GWAS, the significant co-localizations we describe implicate *PARP12* and *CLU* as new candidate genes for these disorders.

Among the four genes exceeding our threshold for eQTL and AMD GWAS colocalization, *RDH5*, encoding a retinol dehydrogenase that catalyzes the conversion of 11-cis retinol to 11-cis retinal in the visual cycle^66^, showed the most significant signal (Fig. 5a and Supplementary Data 11). *RHD5* was previously suggested as an AMD candidate gene^42^, but no mechanism was proposed. Two tightly linked AMD-associated variants (rs3138141 and rs3138142, *r*^2^ = 0.98) are highly correlated with *RDH5* expression (Fig. 5b). The minor haplotype identified by the rs3138141 “a” allele is associated with a significantly smaller percentage of total *RDH5* expression (26.4%) than the major haplotype identified by the “C” allele (73.6%) (Fig. 5g). We found no evidence for an effect on transcripts from the adjacent *BLOC1S1* gene or on *BLOC1S1-RDH5* read-through transcripts. The same variants mark an *RDH5* sQTL (Fig. 5a, c) associated with differences in the usage of exon 3 of the transcript; samples that are heterozygous at rs3138141 (Ca) exhibit an average of more than three times the amount of exon 3 skipping compared to CC homozygous samples (Fig. 5h and Supplementary Fig. 8). The same e/sQTL also colocalized with a myopia GWAS signal (Fig. 5d–f, Supplementary Data 12), suggesting a mechanism for the prior association of the *RDH5* locus with myopia^67^ and refractive error^13^.

### NMD as a putative mechanism underlying an RDH5 eQTL

The association of the rs3138141/2 minor haplotype with both an *RDH5* eQTL and sQTL suggests a mechanistic relationship. We estimate that ~80% of isoforms transcribed from the “C” haplotype are normal, whereas ~75% of isoforms transcribed from the “a” haplotype are mis-spliced (see Methods section). The increased skipping of exon 3 (out of 5) associated with the minor haplotype results in more transcripts with a frameshift and a premature termination codon (PTC) near the 5′ end of exon 4. Many mammalian transcripts with PTCs are subject to NMD, particularly when the PTC is not located in the last exon^68^. Treatment of cells with protein synthesis inhibitors such as cycloheximide (CHX) has been shown to increase the abundance of transcripts subject to NMD^69^. To assess a possible role for NMD in the stability of RDH5 transcripts, we treated differentiated immortalized human RPE cells (ARPE-19) with CHX and quantified the abundance of the normal and skipped exon 3 isoforms by RT-PCR. CHX caused a significant increase in the abundance of the skipped exon 3 isoform as compared to the normal (Fig. 5i–k and Supplementary Fig. 9). These data are consistent with a model in which the minor allele promotes the formation of an aberrant RDH5 mRNA that is subject to NMD, leading to an overall reduction in the steady state levels of RDH5 transcripts.

## Discussion

The importance of the RPE for development and lifelong homeostasis of the eye has motivated numerous studies of the RPE transcriptome. Several of these studies proposed similar sets of RPE “signature” genes, the largest of which comprises 171 genes^29–31^. Only 23 of these genes are present among our group of 100 fRPE-selective protein-coding genes. Our approach of comparing fRPE expression levels to GTEx data, which almost exclusively derive from adult autopsy tissue specimens, may have captured genes highly expressed in cultured and/or fetal cells. Absence in GTEx of pure populations of specialized cell types, especially ocular, may explain other genes in our set. Still, many of the genes we identified are known to serve vital functions in the RPE, as demonstrated by pathway enrichment for pigment synthesis and visual processes. We also identified 30 enriched lncRNAs, a class of transcripts not included in previous signature gene sets. The most highly expressed lncRNA in our list, *RMRP*, is critical for proper mitochondrial DNA replication and OXPHOS complex assembly in HeLa cells^70^, but its role in the RPE has not yet been investigated. RPE-enriched genes whose functions have not been studied in the tissue afford opportunities for advancing understanding of this important epithelial layer.

Our findings have potential implications for phenotypic variability in monogenic ocular diseases. Mutations in all three of the fRPE-selective eGenes cause monogenic eye diseases. For example, heterozygous mutations in the transcription factor *CRX* cause dominant forms of photoreceptor degeneration, which can exhibit variable age at onset and disease progression among members of the same family^4^. Genetically encoded variation in the transcript levels of normal or mutant *CRX* alleles may contribute to such variable expressivity. Indeed, mouse models of *CRX*-associated retinopathies provide evidence for a threshold effect in which small changes in expression cause large differences in phenotype^71^. Mutations in *MRFP* cause extreme hyperopia (farsightedness). Affected individuals usually have two mutant alleles, but inheritance of a lower-expressing normal allele could explain an affected heterozygous individual in a family with otherwise recessive disease^5^. The substantial number of fRPE eQTLs associated with other ocular diseases (Fig. 3a) supports a contribution of common genetic variants to the widespread phenotypic variability observed in monogenic eye disorders.

Our findings also have implications for complex ocular diseases. Evidence suggests that defects in RPE energy metabolism contribute to the pathogenesis of AMD, the hallmark of which is accumulation of cholesterol rich deposits in and around the RPE^72,73^. Forcing fRPE cells to rely on oxidation of glutamine, the most abundant free amino acid in blood, caused upregulation of genes involved in the synthesis of cholesterol, monounsaturated and polyunsaturated fatty acids, as well as genes associated with lipid import. Transcripts for three of the upregulated genes (*FADS1*, *FADS2*, and *ACAT2*) are increased in macular but not extramacular RPE from individuals with early-stage AMD^74^.

Co-localization of the same *RDH5* e/sQTL with both AMD and myopia GWAS loci suggests risk mechanisms for these very different complex diseases. The rs3138141/2 minor haplotype confers an elevated risk for AMD^42^, but is protective for myopia^13,43,67^. Reduction in RDH5 activity as a risk factor for AMD is consistent with rare *RDH5* loss-of-function mutations that cause recessive fundus albipunctatus, which can include macular atrophy^75,76^. More puzzling is the relationship between lower RDH5 transcript levels (and presumably enzyme activity) and a reduced risk of myopia. RDH5 is best known for its role in the regeneration of 11-cis retinal in the visual cycle, but the enzyme has also been reported to be capable of producing retinoids suitable for retinoic acid signaling^77,78^. Evidence from animal models implicates retinoic acid in eye growth regulation^12^, and retinal all-trans retinoic acid levels are elevated in a guinea pig model of myopia^79^. Thus the same allele, which has risen to substantial frequencies in some populations (0.38 minor allele frequency in South Asians and 0.19 in Europeans https://www.ncbi.nlm.nih.gov/projects/SNP/), may dampen retinoic acid signaling during eye development and growth, and later contribute to chronic photoreceptor dysfunction in older adults.

The eye is a highly specialized organ with limited representation in large-scale functional genomics datasets. Our analysis of genetic variation and metabolic processes in fRPE cells, even with modest sample sizes, expands our ability to map functional variants with potential to contribute to complex and monogenic eye diseases. Future studies with larger sample sizes from geographically diverse populations, and/or targeting other ocular cell types, will likely discover additional e/sQTLs and functional variants involved in genetic eye diseases.

## Methods

### Sample acquisition and cell culture

Primary human fetal RPE (fRPE) lines were isolated from fetal eyes (Advanced Biosciences Resources, Inc., Alameda, CA) by collecting and freezing non-adherent cells cultured in low calcium medium as described^22^. When needed, fRPE cells were thawed and plated onto 6-well plates in medium as described^23^ with 15% FBS. The next day, medium was changed to 5% FBS and the cells were allowed to recover for two additional days. Cells were then trypsinized in 0.25% Trypsin-EDTA (Life Technologies Corporation), resuspended in medium with 15% FBS and plated onto human extracellular matrix-coated (BD Biosciences) Corning 12-well transwells (Corning Inc., Corning, NY) at 240 K cells per transwell. The next day medium was changed to 5% FBS. Cells were cultured for at least 10 weeks to become differentiated (transepithelial resistance of >200 Ω * cm^2^) and highly pigmented. Medium with 5% FBS was changed every 2–3 days. For the galactose and glucose specific culture conditions, differentiated fRPE cells were cultured for 24 h prior to RNA isolation in DMEM medium (Sigma) with 1 mM sodium pyruvate (Sigma), 4 mM l-glutamine (Life Technologies Corporation), 1% Penicillin-Streptomycin (Life Technologies Corporation), and either 10 mM d-(+)-glucose (Sigma) or 10 mM d-(+)-galactose (Sigma)^28^. The fRPE lines studied here are not available for distribution.

### Genotype data and quality control

#### Microarray library preparation and genotyping

All 24 RPE samples were genotyped on three Illumina Infinium Omni2.5-8 BeadChip using the Infinium LCG Assay workflow (https://www.illumina.com/products/by-type/microarray-kits/infinium-omni25-8.html). A total of 200 ng of genomic DNA was extracted and amplified to generate sufficient quantity of each individual DNA sample. The amplified DNA samples were fragmented and hybridized overnight on the Omni2.5-8 BeadChip. The loaded BeadChips went through single-base extension and staining, and were imaged on the iScan machine to obtain genotyping information. Genotyping data were exported from Illumina GenomeStudio to ped and map pairs, merged, and converted to the VCF format using PLINK v1.9^80^. We removed variants that were missing in more than 5% of samples.

#### Variant annotation

We annotated variants using genomic features (including downstream-gene variant, exonic variant, intronic variant, missense variant, splice-acceptor variant, splice-donor variant, splice-region variant, synonymous variant, upstream-gene variant, 3′-UTR variant, 5′-UTR variant), loss-of-function, and nonsense-mediated decay predictions, and clinical databases (including ClinVar, OMIM and OrphanNet) using SnpEff v4.3i^81^.

#### Imputation and phasing

We used Beagle v4.1^82^ to perform genotype imputation and phasing. Genotypes were imputed and phased with 1000 Genomes Project phase 3 reference panel. Before imputation and phasing, we filtered the original VCF file to only bi-allelic SNP sites on autosomes and removed sites with more than 5% missing genotypes. We also re-coded the VCF file based on the reference and alternative allele designation of 1000 Genomes Project phase 3 reference panel using the conform-gt program which was provided with the Beagle software.

#### Quality control

Prior to imputation, we performed standard pre-imputation QC by removing variants that are missing in more than 5% of samples and used the filtered call set as input to Beagle. After imputation and phasing, we removed variants with allelic *r*^2^ < 0.8 (higher allelic *r*^2^ indicates higher confidence). Standard post-imputation QC also requires removing variants with low Hardy–Weinberg Equilibrium (HWE) *p*-value. Due to the extensive admixture in our study cohort, we reasoned that HWE may have trouble in distinguishing genotyping error from admixture. We opted to not apply HWE but instead to remove multi-allelic variants (variants with more than two alleles). The Ts/Tv ratio of the filtered call set was above 2.0 for all chromosomes but chromosome 8 (=1.98) and 16 (=1.93). Several chromosomes have Ts/Tv ratio greater than 2.1, indicating that they are enriched with known (vs. novel) variants (Supplementary Fig. 10). To detect sample duplication, we plotted genotype correlation across every pair of samples and found two samples (sample 3 and 5) were duplicates of each other (Supplementary Fig. 11). We removed one sample (sample 5) at random. The filtered VCF files were used for downstream analysis.

#### Sex determination

We determined biological sex of each donor using genotyping information. We extracted the genotype dosage with bcftools and calculated the proportion of heterozygous SNPs (heterozygous: dosage = 1; homozygous: dosage = 0 or 2) for chromosome 1 and X. Donors with low heterozygosity on chromosome X (proportion of heterozygous ≈ 0) SNP were defined as males. Chromosome 1 was used as control to establish the baseline for heterozygosity. This cohort has 11 male and 13 female individuals (Supplementary Fig. 12).

#### Ancestry determination

We determined ancestry of each donor using genotype information. We extracted genotype dosages of chromosome 1 for 23 RPE samples and 4 samples from each of the 26 populations (for a total of 104 individuals) in 1000 Genomes phase 3 version 5 dataset^25^. We calculated the principal components using the prcomp function in R. The top three principal components explained the most variability (Supplementary Fig. 13), and were used for downstream analysis. The first two principal components clearly separates the European, African, and Asian populations. Four RPE samples are European, and the rest are admixed. Most admixed individuals are African American (Supplementary Fig. 1).

### Transcriptomic data and quality control

#### RNA-seq library preparation and sequencing

RNA was extracted using TRIzol Reagent (Invitrogen) per manufacturer instructions. RNA sequencing was performed on all samples with an RNA integrity number (RIN) of 8.0 or higher and with at least 500 ng total RNA. Stranded, poly-A+ selected RNA-seq libraries were generated using the Illumina TruSeq Stranded mRNA protocol. We performed 75 bp paired-end RNA sequencing on an Illumina NextSeq 500 on all RPE samples (Supplementary Data 2). Glucose and galactose samples from each line were sequenced together to minimize batch effects.

#### RNA sequencing read mapping

Raw data was de-multiplexed using bcl2fastq2 from Illumina with default parameters. Reads were aligned against the hg19 human reference genome with STAR (v2.4.2a)^83^ using GENCODE v19 annotations^84^ and otherwise default parameters. After alignment, duplicate reads were marked using Picard MarkDuplicates (v2.0.1) and reads marked as duplicates or with non-perfect mapping qualities were removed.

#### Gene and splicing event quantification

We used HTSeq v0.6.0^85^ to count the number of reads overlapping each gene based on the GENCODE v19 annotation. We counted reads on the reverse strand (ideal for Illumina’s TruSeq library), required a minimum alignment quality of 10, but otherwise used default parameters. We also quantified RPKM using RNA-SeQC v1.1.8^86^ using hg19 reference genome and GENCODE v19 annotation with flags “-noDoC -strictMode” but otherwise default parameters. We quantified allele-specific expression using the createASVCF.sh script from RASQUAL^45^ with default parameters. For splicing quantification, we used LeafCutter^59^ to determine intron excision levels with default parameters. Briefly, we first converted bam files to splice junction counts (bam2junc.sh) and clustered introns based on sharing of splice donor or acceptor sites (leafcutter cluster.py). For each cluster, We required a minimum number of 30 reads, and a minimum fraction of 0.1% in support of each junction. We required each intron must not exceed 100 kbp.

#### Quality control

We profiled the RNA-seq library to a median depth of 52.7 million reads (interquartile range: 45.5–60.1 million reads), for a total of 2.5 billion reads (Supplementary Fig. 14a). We checked the number of uniquely mapped reads to ensure sufficient number of mapped reads. The RNA-seq libraries have a median number of 46.8 million (88.8%) uniquely mapped reads, with an interquartile range of 41.0–55.2 million reads (Supplementary Fig. 14b). We ran VerifyBamID^87^ with parameters “—ignoreRG—best” on RNA-seq BAM files using genotype VCF files as reference and did not find any sample swaps.

#### Normalization of quantifications

We extract hidden factors from RNA sequencing data using surrogate variable analysis (sva)^88^ jointly (protecting the treatment variable) and separately for glucose and galactose-treated samples. Prior to estimating hidden factors, the raw count gene expression data was library size corrected, variance stabilized, and log2 transformed using the R package DESeq2^34^. Genes with average read count below 10 and with zero counts in more that 20% of samples were considered not expressed and filtered (to remove tails). A total of 15,056 and 15,062 expressed genes remained for glucose and galactose, respectively. Since library size correct depends on all genes, filtered genes were again corrected for library size, variance stabilized, and log2 transformed using DESeq2 before being used as input to sva. We ran sva, as implemented in the sva R package, with default parameters, and obtained seven significant surrogate variables with joint analysis and four and five significant surrogate variables for the glucose and galactose condition, respectively. We also extracted surrogate variables for splicing level quantification. The joint, glucose, and galactose analysis returned four, two, and two factors, respectively.

#### Correlation between known and hidden confounders

We calculated the correlation between known (treatment, RIN, sequencing batch, sex, and ancestry) and hidden (surrogate variables) factors to determine which factors to include in downstream analyses. The jointly inferred factors (seven in total) captures treatment (factor 5, *r* = 0.91), RIN (factor 1, *r* = 0.71) and batch effect (factor 7, *r* = −0.56), but does not capture sex (best *r* = −0.18) or ancestry (best *r* = −0.34). This agrees with the intuition that treatment, RIN and batch effect have broad influences on gene expression measurement, while sex and ancestry only influence a small set of relevant genes (Supplementary Fig. 15a). To reduce the correlation between factor 5 and treatment, we ran supervised sva protecting the treatment effect. Even with protection on treatment, factor 5 remains correlated (*r* = −0.62), likely due to the strong and broad effect exerted by metabolic perturbation (Supplementary Fig. 15b). The glucose surrogate variables captured RIN (factor 1, *r* = 0.81) and batch (factor 2, *r* = 0.78), and the galactose surrogate variables captured RIN (factor 1, *r* = −0.61) and batch (factor 1 and 4, *r* = −0.54 and −0.5, respectively). None of the glucose or galactose surrogate variables captured sex or ancestry (Supplementary Figs. 15c, d). We also compared surrogate variables from splicing quantification. Without protecting the treatment, surrogate variable 3 from the joint set correlated with the treatment (Supplementary Fig. 16a). Even after protecting the treatment, surrogate variable 1 from the joint set correlated with the treatment (Supplementary Fig. 16b), similar to what was observed for expression surrogate variables. Surrogate variables from the glucose and galactose condition correlated strongly with RIN (*r* = −0.74 and −0.76, respectively, Supplementary Fig. 16c, d).

### External datasets

#### RNA-seq and eQTL datasets

We used GTEx V7^18^ as a reference dataset to perform RPE-selective gene and RPE-specific eQTL analyses. The GTEx V7 dataset collected 53 tissues across 714 donors. All tissues across all donors were used in RPE-selective gene analysis. Among the 53 tissues, 48 tissues have sufficient sample size to perform eQTL analysis and were used for RPE-specific eQTL calling.

#### GWAS datasets

We used two well-powered ocular disorder GWAS datasets to perform colocalization analyses. The AMD study^42^ is a meta-analysis across 26 studies and identified 52 independent GWAS signals, including 16 novel loci. The myopia GWAS was part of a 42-trait GWAS collection aimed at finding shared genetic influences across different traits^43^.

#### Ocular disease genes dataset

We used ocular disease genes from the Genetic Eye Disease (GEDi) test panel^41^, which encompasses 257 genes in total including known inherited retinal disorder genes (IRD, *n* = 214), glaucoma and optic atrophy genes (*n* = 8), candidate IRD genes (*n* = 24), age-related macular degeneration risk genes (*n* = 9), and a non-syndromic hearing loss gene (*n* = 1).

### RPE-selective gene and pathway enrichment analyses

#### Expression *z*-score method

To identify RPE-selective genes (high expression in RPE relative to other tissues), we inferred expression specificity using the following procedure.

1. Calculate the median expression level (*x*) across all individuals for each tissue.
2. Calculate the mean (*μ*) and standard deviation (*σ*) of median expression values across tissues.
3. Derive a *z*-score for each tissue as follows: *z* = (*x*−*μ*)/*σ*.
4. Define a gene to be tissue-selective if its *z*-score is greater than 4.

We filtered out genes on sex and mitochondrial chromosomes, and further filtered out genes in HLA region due to low mappability. To determine whether technical confounders (such as batch effect) affected RPE *z*-scores, we used a QQ-plot to visualize the *z*-score of each tissue against the average *z*-score across tissues. To calculate the average *z*-scores, we ranked genes within each tissue and take the average *z*-score for genes with the same rank across tissues. The average *z*-scores represent the expected distribution. If the *z*-score distribution from a tissue is markedly different from the expected distribution, this distribution will separate from the diagonal on the QQ plot. Supplementary Fig. 17 shows that RPE *z*-scores situate within the midst of *z*-scores from GTEx tissues. In fact, the only outlier is testis, which is a known outlier from previous studies.

#### Pathway enrichment of RPE-selective genes

To identify coordinated actions by RPE-selective transcriptomic elements, we performed GSEA^32^ using *z*-scores as input against GO gene sets from the Molecular Signature Database^89^ with 10,000 permutations and otherwise default parameters. The full results are in Supplementary Data 4.

### Differential expression and pathway enrichment analyses

#### Differential expression analysis

We performed differential expression analysis with DESeq2^34^ to detect genes whose expression levels were affected by metabolic perturbation. Due to the correlation between hidden factors (SVs) and treatment, we decided to use known factors in the DESeq2 model. More specifically, we observed moderate correlation between SVs and condition (*r* = −0.62) even after protecting for condition (Supplementary Fig. 15). Adding SVs that are correlated with conditions will bias the estimates on the coefficient for conditions. The model is shown below:

$${E({\mathrm{expression}}) = \beta _0 + \beta _t \cdot {\mathrm{treatment}} + \beta _s \cdot {\mathrm{sex}} + \mathop {\sum }\limits_{i = 1}^3 \beta _{a,i} \cdot {\mathrm{PC}}_i + \beta _r \cdot {\mathrm{RIN}} + \beta _b \cdot {\mathrm{batch}}}$$

#### Pathway enrichment analysis

We performed pathway enrichment analysis with GSEA using a ranked gene list with 10,000 permutations but otherwise default parameters. The ranking metric was calculated by multiplying the −log_10_(FDR) by the sign of the effect size from DESeq2. For the pathway database, we used a subset of the Molecular Signatures Database composed of its Hallmark, Biocarta, Reactome, KEGG and GO gene sets^89^.

### fRPE-selective genes in ocular diseases

#### RPE-selective expression in ocular disease genes

We stratified all protein-coding genes into two groups: (1) ocular disease genes (*n* = 257) and (2) non-ocular disease genes (*n* = 18,477). To determine whether known ocular disease genes have elevated expression in fRPE, we compared the expression specificity *z*-score distribution (defined previously) across these two groups with a two-sided *t*-test. We performed the same analysis for all GTEx tissues as a benchmark. As a control, we repeated this analysis using known epilepsy genes (*n* = 189) curated from the Invitae epilepsy gene test panel (https://www.invitae.com/en/physician/tests/03401/).

#### RPE-selective expression in ocular disease GWA studies

GWAS risk loci are frequently enriched around causal genes, which have elevated expression in relevant tissues^90^. To determine whether variants around RPE-selective genes explain higher disease heritability than expected by chance, we performed stratified LD score regression on tissue-selective genes using a previously established pipeline^44^. Since LD score regression operates on a variant level, we assigned variants within 1-kb around any exon of tissue-selective genes to each tissue. Although many variants show long-range interaction, we restricted our analysis to a conservative window size to capture only nearby cis-effects. We performed LD score regression on the 200, 500, and 1000 tissue-specific genes (Fig. 3 and Supplementary Fig. 3).

### eQTL mapping and quality control

#### Covariate selection

We determined biological sex, genomic ancestry, and hidden confounders as described in previous sections. We performed covariate selection by empirically maximizing the power to detect eQTL. We randomly selected 50 genes from chromosome 22 to perform covariate selection for computational feasibility and to avoid overfitting. We added sex, genotype principal components (maximum of three), and surrogate variables sequentially. We chose not to include batch effect or RIN because they were well represented by surrogate variables. We tested the top three genotype principal components because they explained most of the variability in the genotyping data (Supplementary Fig. 13). After multiple hypothesis correction, the number of eAssociations (defined as a SNP-gene pair that passed hierarchical multiple hypothesis testing by TreeQTL^46^) increased monotonically for both glucose and galactose conditions as the number of covariates increased (Supplementary Fig. 18), which agrees with our intuition that sva only returns significant and independent surrogate variables. Therefore, we decided to use sex, top three genotype principal components and all surrogate variables (four and five for glucose and galactose conditions, respectively).

#### Per-treatment eQTL calling

We mapped eQTL using RASQUAL^45^, which integrates total read count with allele-specific expression (ASE) to boost power for eQTL mapping. To obtain GC-corrected library size, We first calculated GC content using GENCODE v19^84^ by taking the average GC content of all exons of a given gene. Next, we calculated GC-corrected library sizes were calculated based on read count output from HTSeq v0.6.0^85^. We used sex, ancestry principal components, and all surrogate variables. Mathematically, the model is the following:

$$E\left( {{\mathrm{expression}}} \right) = \beta _0 + \beta _{\mathrm{g}} \cdot {\mathrm{genotype}} + \beta _{\mathrm{s}} \cdot {\mathrm{sex}} + \mathop {\sum }\limits_{i = 1}^3 \beta _{a,i} \cdot {\mathrm{PC}} + \mathop {\sum }\limits_{i = 1}^n \beta _{{\mathrm{s}},i} \cdot {\mathrm{SV}}$$

where *e* stands for expressions, *g* stands for genotypes, PC stands for genotype principal components, SV stands for surrogate variables, and *n* = 4 and 5 for glucose and galactose conditions, respectively. We obtained gene-level and association-level FDR using a hierarchical hypothesis correction procedure implemented in TreeQTL^46^. TreeQTL uses a hierarchical FDR correction procedure, which performs FDR correction first on the gene level, and then on the association level (gene by SNP). We used FDR < 0.05 on both gene and association levels.

#### eQTL quality control

We determined whether the *p*-values were inflated (e.g., due to model mis-specification) by visualizing their distribution. The distribution suggests that the p-values are slightly conservative. The spike around zero and upward trend in the QQ plot shows clear enrichment for significant eQTL (Supplementary Fig. 19a, b). As expected, eQTLs with low p-values were enriched around transcription start sites (Supplementary Fig. 19c, d).

#### Differential eQTL calling with TreeQTL

We performed multi-tissue eQTL calling, using the RASQUAL *p*-values and the multi-tissue version of TreeQTL^46^. We set the gene as the first level, the treatment as the second level, and the gene-treatment-SNP as the third level and used the default FDR < 0.05 cutoff for all three levels. We showed a comparison of −log_10_(*p*-value) across two metabolic conditions in Supplementary Fig. 4, in which the top five treatment-specific and shared eQTL are labeled. We ranked the differential eQTL result in the order of decreasing δ|*π* − 0.5| (difference in allelic imbalance in two conditions). More specifically, *π* denotes the allelic ratio (alternative allele/reference allele), and |*π* − 0.5| denotes the allelic imbalance. The difference in allelic imbalance, δ|*π* − 0.5|, defines the change in eQTL effect size across two conditions. We show the allelic ratios in Supplementary Fig. 5.

#### RPE-selective eQTL

We compared RPE and GTEx eGenes with a two-step FDR approach as described previously^56^. In brief, eGenes shared across both conditions in fRPE were selected (FDR < 0.05). We decided to filter for shared eGenes because they likely reflect regulatory effects not due to treatments. For each eGene, we screened all GTEx tissues for association at a relaxed FDR < 0.1 and defined an eGene as RPE-selective if no significant association were found in GTEx. Note that such strategy is conservative on two levels. First, by selecting shared eQTL in RPE, these eQTL must pass FDR < 0.05 in both treatments. Second, GTEx FDR corrections were performed tissue-by-tissue, and per-tissue FDR is anti-conservative. We also compared fRPE eGenes to retinal eGenes. The EyeGEx dataset used 406 samples to map eQTLs in the retina and found a total of 10,463 eGenes. We again selected eGenes found in both glucose and galactose conditions (*n* = 687) and grouped them into fRPE-specific and EyeGEx-shared if they were also eGenes in the EyeGEx dataset.

#### Motif enrichment in treatment-specific eQTLs

In order to find motifs enriched around treatment-specific eQTLs, we first selected the lead eQTLs from either condition and extracted the 15 bp flanking the lead SNP as the target sequences. To obtain matched background sequence, we flipped the eQTL SNP to its alternative allele as the background. To keep the direction of effect consistent, we always used the expression-increasing allele as the target and the expression-decreasing allele as the background. The target and background sequences were used as input to HOMER to identify enriched motifs.

### sQTL calling and quality control

#### Covariate selection

We performed covariate selection by empirically maximizing the power to detect sQTL. We used intron clusters only from chromosome 1 to avoid overfitting, and tested only the top three genotype principal components because they explained most of the variability in the genotyping data (Supplementary Fig. 13). FastQTL were run in permutation mode (adaptively permute 100–10,000 times) to obtain intron-level sQTL *p*-values. After multiple hypothesis correction, the number of significant sQTL cluster decreased as the number of covariates increased (Supplementary Fig. 20). This is likely because LeafCutter uses the ratio between each intron and its intron cluster as the phenotype. Suppose a batch effect influences the expression of a gene. Such batch effect will influence the quantification of each intron in the same direction. Taking the ratio between a intron and its intron cluster effectively cancels out the batch effect.

#### Per-treatment sQTL calling

We mapped sQTLs separately for two conditions using FastQTL^60^ in both nominal and permutation modes and used a simple linear regression:

$$E({\mathrm{intron}}) = \beta _0 + \beta _{\mathrm{g}} \cdot {\mathrm{genotype}}$$

where *s* stands for the ratio between reads overlapping each intron and the total number of reads over-lapping the intron cluster, g stands for genotypes. To obtain cluster-level *p*-values, we used a conservative approach to correct for family-wise error rate with the Bonferroni procedure across introns within each cluster. Global FDR estimates were calculated using the lowest Bonferroni adjusted *p*-values per cluster. We used FDR < 0.05 as a significance cutoff.

#### sQTL quality control

As a quality control, we determined whether the *p*-values were inflated by visualizing their distribution. The *p*-values showed a uniform distribution with a spike near 0 (Supplementary Fig. 21a). The upward trend in the QQ plot shows clear enrichment for significant eQTL (Supplementary Fig. 21b). Further, sQTLs with low *p*-values were enriched around splicing donor and acceptor sites (Supplementary Fig. 21c), and intronic sQTL SNPs were enriched at intron boundaries (Supplementary Fig. 21d).

### Fine-mapping of polygenic ocular disease risk loci

We used fRPE eQTL and sQTL information to identify potential causal genes in two well-powered GWAS on age-related macular degeneration and myopia using a modified version of eCAVIAR^65^. For every significant eQTL, we tested all variants within 500-kb of the lead eQTL SNP for colocalization with GWAS summary statistics. At each candidate locus, we ran FINEMAP^91^ twice to compute the posterior probability that each individual SNP at the locus was a causal SNP for the GWAS phenotype and fRPE e/sQTLs. We then processed the FINEMAP results to compute a colocalization posterior probability (CLPP) using the method described by eCAVIAR^65^. We defined any locus with CLPP > 0.01 to have sufficient evidence for colocalization. At loci that showed colocalization between RPE eQTLs and GWAS associations, we performed the colocalization tests again using eQTLs from each of 44 GTEx tissues. To determine whether any potential causal genes act primarily through fRPE, we repeated colocalization analysis with GTEx eQTLs (Supplementary Figs. 22 and 23). To identify condition-specific colocalization, we ran eCAVIAR separately for the glucose and galactose conditions. A condition-specific colocalization is defined as having a CLPP > 0.01 in one condition, and at least an order of magnitude lower in the other condition with a CLPP < 0.01.

### Estimation of isoform proportions

To estimate the proportions of the normal and mis-spliced isoform (exon-3 skipped isoform), we solved a system of equations based on the following observations:

The “C” haplotype produces approximately 3 times as much as the “a” haplotype.

1. The mis-spliced isoform accounts for approximately 1% of expression in individuals with CC genotype.
2. The mis-spliced isoform accounts for approximately 4% of expression in individuals with Ca genotype.

We use *n*_c_ and *n*_a_ to denote the proportion of normal isoform for the “C” and the “a” haplotypes, and *p*_n_ and *p*_m_ to denote the proportion of normal and mis-spliced isoforms that pass non-sense mediated decay (not degraded). For simplicity, we assume that *p*_n_ = 1 because normal isoform should not be degraded by NMD. We use *c*_t_, *c*_n_, and *c*_m_ to denote the total, normal, and mis-spliced isoforms for the “C” haplotype, and *a*_t_, *a*_n_, and *a*_m_ to denote the total, normal, and mis-spliced isoforms for the “a” haplotype. We know that:

$$c_{\mathrm{n}} = 100c_{\mathrm{m}}$$

$$c_{\mathrm{n}} + a_{\mathrm{n}} = 25\left( {c_{\mathrm{m}} + a_{\mathrm{m}}} \right)$$

$$c_{\mathrm{t}} = 3a_{\mathrm{t}}$$

Plugging in *c*_n_ = *n*_c_, *c*_m_ = (1 − *n*_c_)*p*_m_, *a*_n_ = *n*_a_ and *a*_m_ = (1 − *n*_a_)*p*_m_:

$$n_{\mathrm{c}} = 100\left( {1 - n_{\mathrm{c}}} \right)p_{\mathrm{m}}$$

$$n_{\mathrm{c}} + n_{\mathrm{a}} = 25\left( {\left( {1 - n_{\mathrm{c}}} \right)p_{\mathrm{m}} + \left( {1 - n_{\mathrm{a}}} \right)p_{\mathrm{m}}} \right)$$

$$n_{\mathrm{c}} + \left( {1 - n_{\mathrm{c}}} \right)p_{\mathrm{m}} = 3\left( {n_{\mathrm{a}} + \left( {1 - n_{\mathrm{a}}} \right)p_{\mathrm{m}}} \right)$$

Solving the system of equations leads to *n*_c_ = 0.82, *n*_a_ = 0.25, and *p*_m_ = 0.05. In other words, 82% and 25% of isoforms transcribed from the “C” and “a” haplotypes are normal, respectively. We estimate that NMD will degrade 95% of mis-spliced isoforms.

### Experimental validation

ARPE-19 cells were obtained from ATCC (CRL-2302). The cells were obtained directly from ATCC within the past year. They exhibit the expected cobblestone morphology and slight pigmentation when differentiated by standard protocols. ARPE-19 cells were fixed, stained with DAPI and imaged by fluorescence microscopy. No evidence of mycoplasma contamination was seen. ARPE-19 cells were differentiated for 3 months in 6-well plates (Corning) in medium containing 3 mM pyruvate^92^ and treated with 100 µg/mL cycloheximide (CHX; Sigma) or vehicle (DMSO) for 3 h. Cells were then collected, RNA was extracted by TRIzol (Invitrogen), and cDNA was synthesized with an iScript™ cDNA Synthesis Kit (Bio-RAD). Oligonucleotide primers were designed to specifically amplify the normal or mis-spliced isoforms of the *RDH5* transcript. For the normal isoform, the forward primer (ggggctactgtgtctccaaa) was located in exon 3 and the reverse primer (tgcagggttttctccagact) was located in exon 4, with an expected product size of 151 bp. The amplification conditions were: 94 °C 2 min followed by 38 cycles of 94 °C 30 s, 60 °C 30 s, 72 °C 15 s. For the mis-spliced isoform, the forward primer (gatgcacgttaaggaagcag/gcg) spanned the exon 2/4 junction with the three bases at the 3′ end located in exon 4. The reverse primer (gcgctgttgcattttcaggt) was located in exon 5. The expected product size is 204 bp. The amplification conditions were: 94 °C 2 min followed by 50 cycles of 94 °C 30 s, 60 °C 30 s, 72 °C 15 s. AmpliTaq (ThermoFisher) and 2.5 mM MgCl_2_ were used for all reactions. The identities of the normal and mis-spliced PCR products were confirmed by Sanger sequencing. For quantification, PCR products were resolved on 2% agarose gels containing ethidium bromide and imaged using a Bio-Rad ChemiDoc Touch Imaging System. Equal-sized boxes were drawn around bands for the CHX and DMSO samples, grayscale values were measured by ImageJ (NIH), and the relative fold change was calculated (mean ± SEM; three independent experiments). A one-sided Students *t*-test was used to assess the statistical significance of a model under which CHX increased product abundance.

### Statistics and reproducibility

To promote the reproducibility of our study, we deposited raw experimental data to GEO (see Data availability) and open sourced all scripts for data processing and analysis (see Code availability).

### Reporting summary

Further information on research design is available in the Nature Research Reporting Summary linked to this article.

## Supplementary information

Supplementary Figures

Description of Additional Supplementary Files

Supplementary Data 1–12

Supplementary Data 13–30

Reporting Summary

## Data Availability

All relevant data are available in the Supplementary Data files (Supplementary Data 1–12). Full eQTL and sQTL summary statistics have been made deposited into Box: https://stanford.box.com/s/asrxy0o66xxe1j7mfj56p3z3d405gijj and are available at http://montgomerylab.stanford.edu/resources.html. RNAseq data can be downloaded via GEO accession number GSE129479. Source data underlying the figures is available as Supplementary Data 13–30.

## Code availability

Code to reproduce all analyses in this manuscript has been deposited on GitHub: https://github.com/boxiangliu/rpe.
