# Supplementary material for: Genetic analyses of human fetal retinal pigment epithelium gene expression suggest ocular disease mechanisms
Source: Commun Biol. 2019 May 20;2:186. doi: 10.1038/s42003-019-0430-6 (PMC6527609; doi:10.1038/s42003-019-0430-6)
Supplement: Supplementary file 1 — Supplementary Figures [file 42003_2019_430_MOESM1_ESM.pdf]

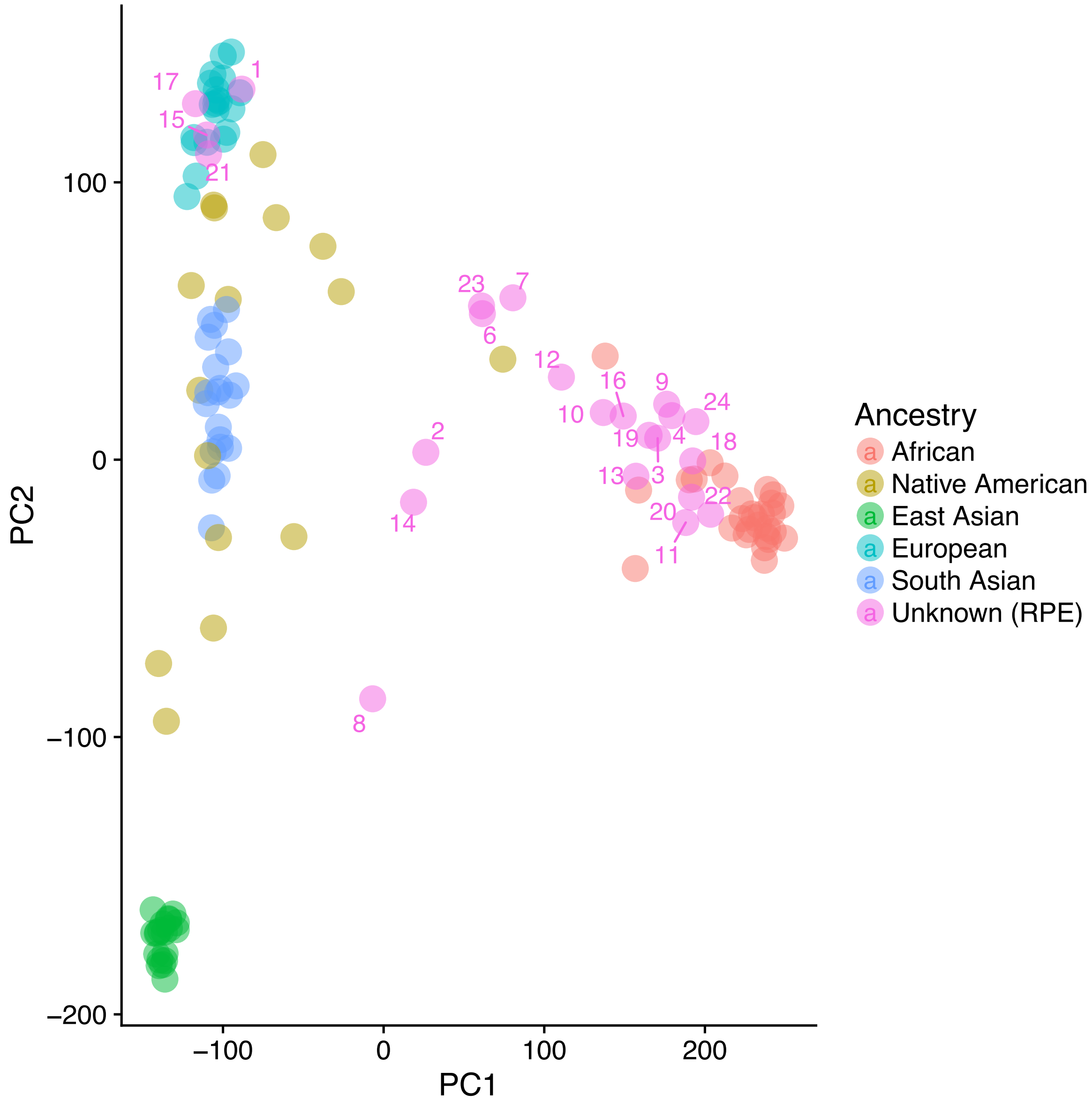

Supplementary Figure 1: Genotype principal components for 1000 Genomes and RPE samples. We obtained genotype principal components using chromosome 1 across 23 RPE samples (duplicate 021011 was removed) and 104 samples from 1000 Genomes phase 3 version 5. Four RPE samples are of European descent, whereas the rest are admixed. Most admixed samples are African American.

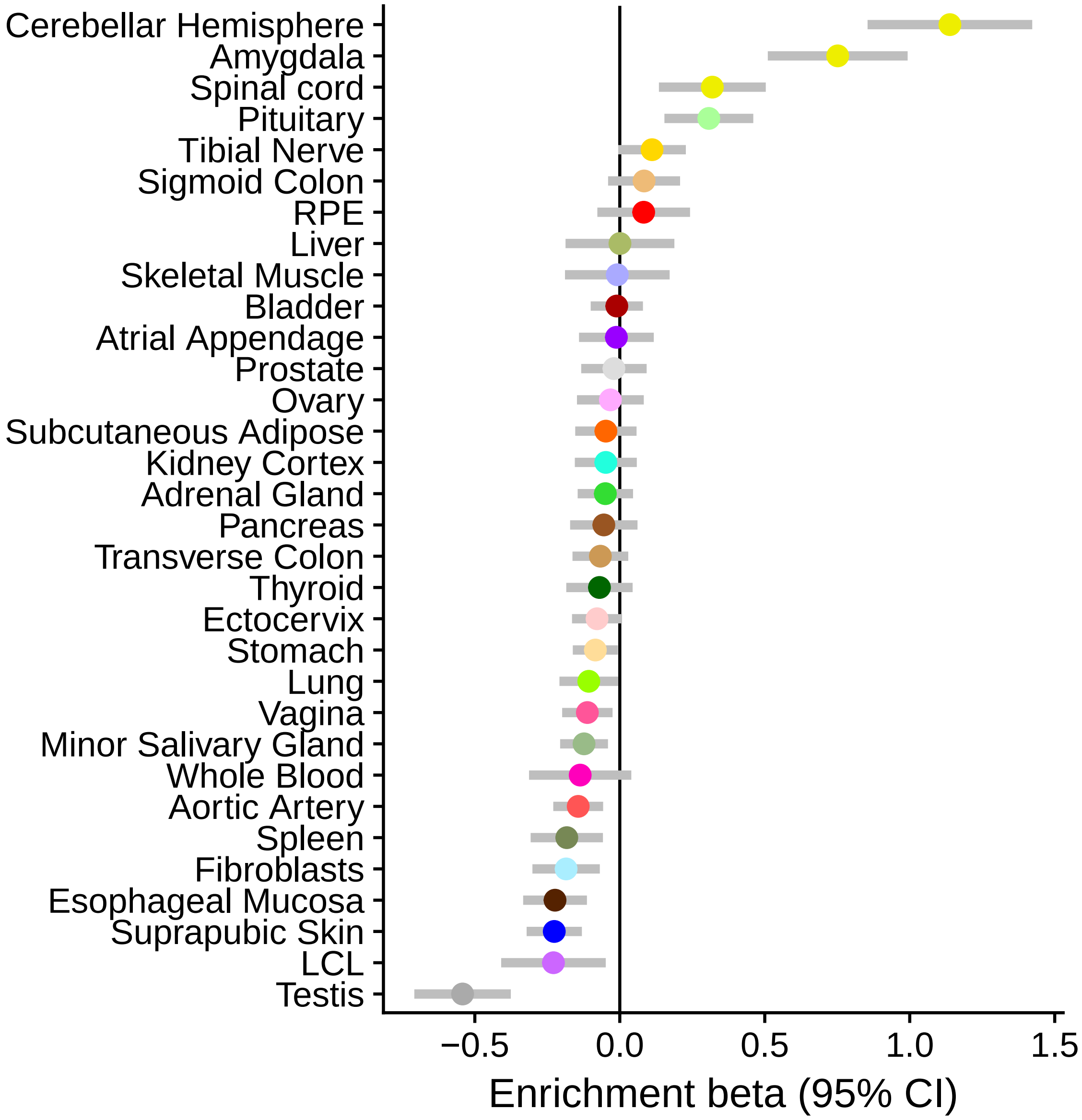

Supplementary Figure 2: Epilepsy genes have elevated expression in brain tissues. As a control, we determined whether epilepsy genes have elevated expression in brain tissues. We obtained a list of known epilepsy genes curated from the Invitae epilepsy gene test panel, and compared the expression of epilepsy genes against other genes. As expected, epilepsy genes have elevated expression in brain tissues.

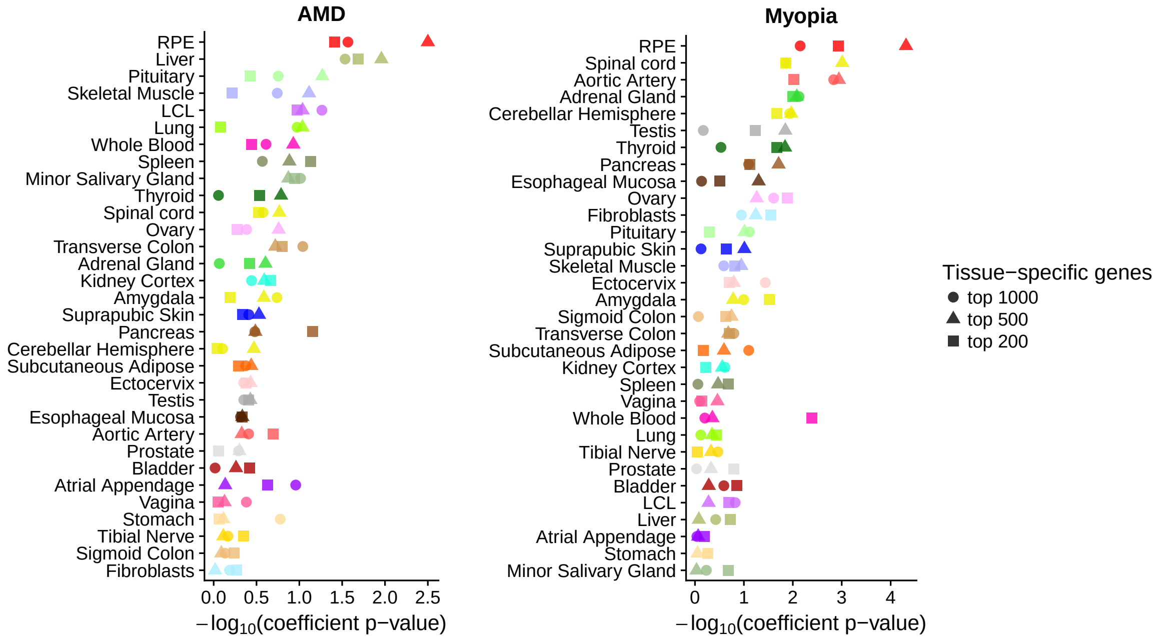

Supplementary Figure 3: LD score regression is robust to selection of tissue-specific genes. We performed stratified LD score regression on top 200, 500, and 1000 genes. The tissue rankings are similar across these three threshold both AMD and Myopia.

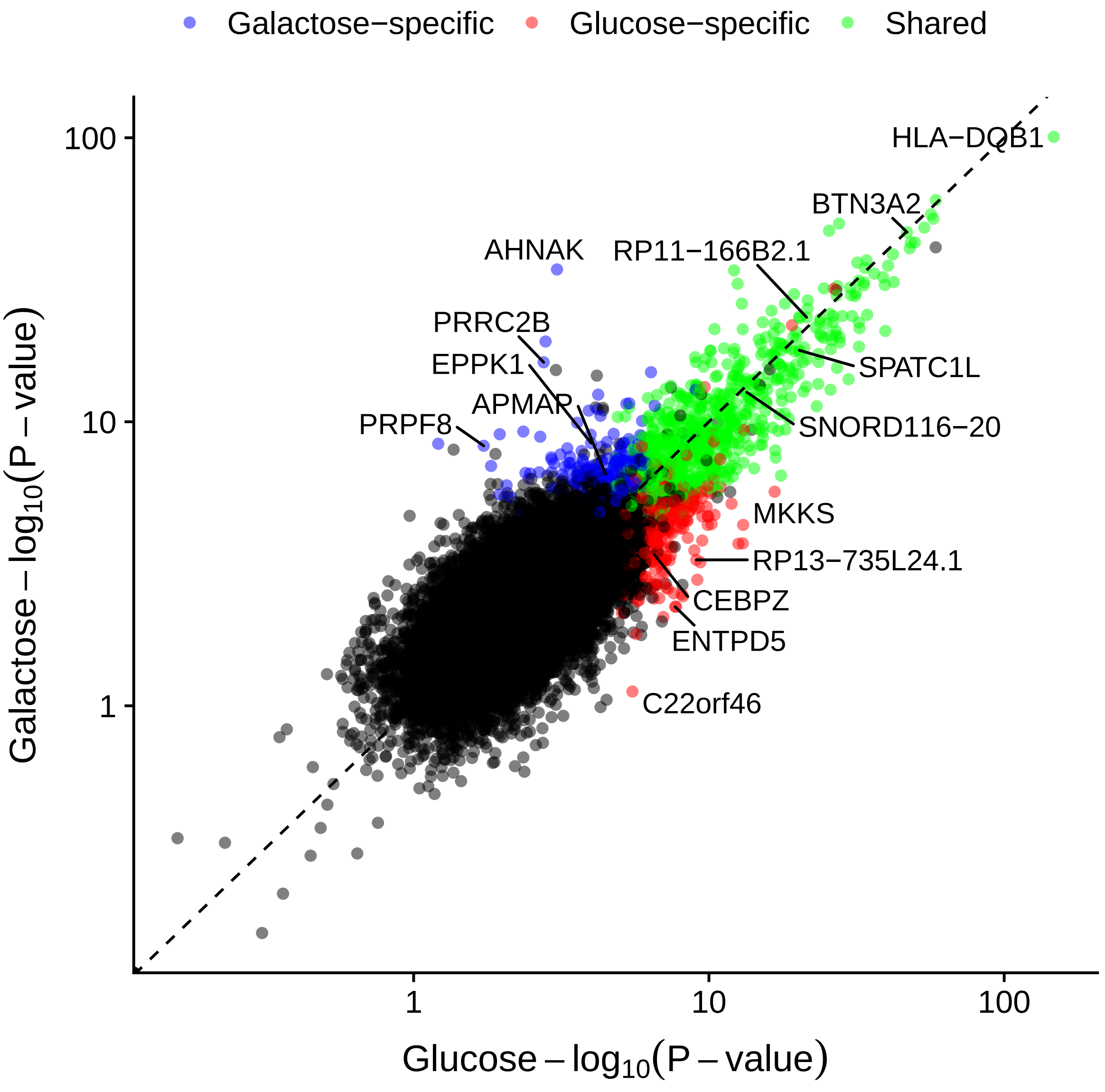

Supplementary Figure 4: Response eQTL. We compared the $-log_{10}(p - value)$ of eQTL across two conditions. The red, blue, and green points indicate glucose-specific, galactose-specific and shared eQTL, respectively. For each class, we labeled the top five eQTL ranked by difference in allelic imbalance across two conditions (section **??**)

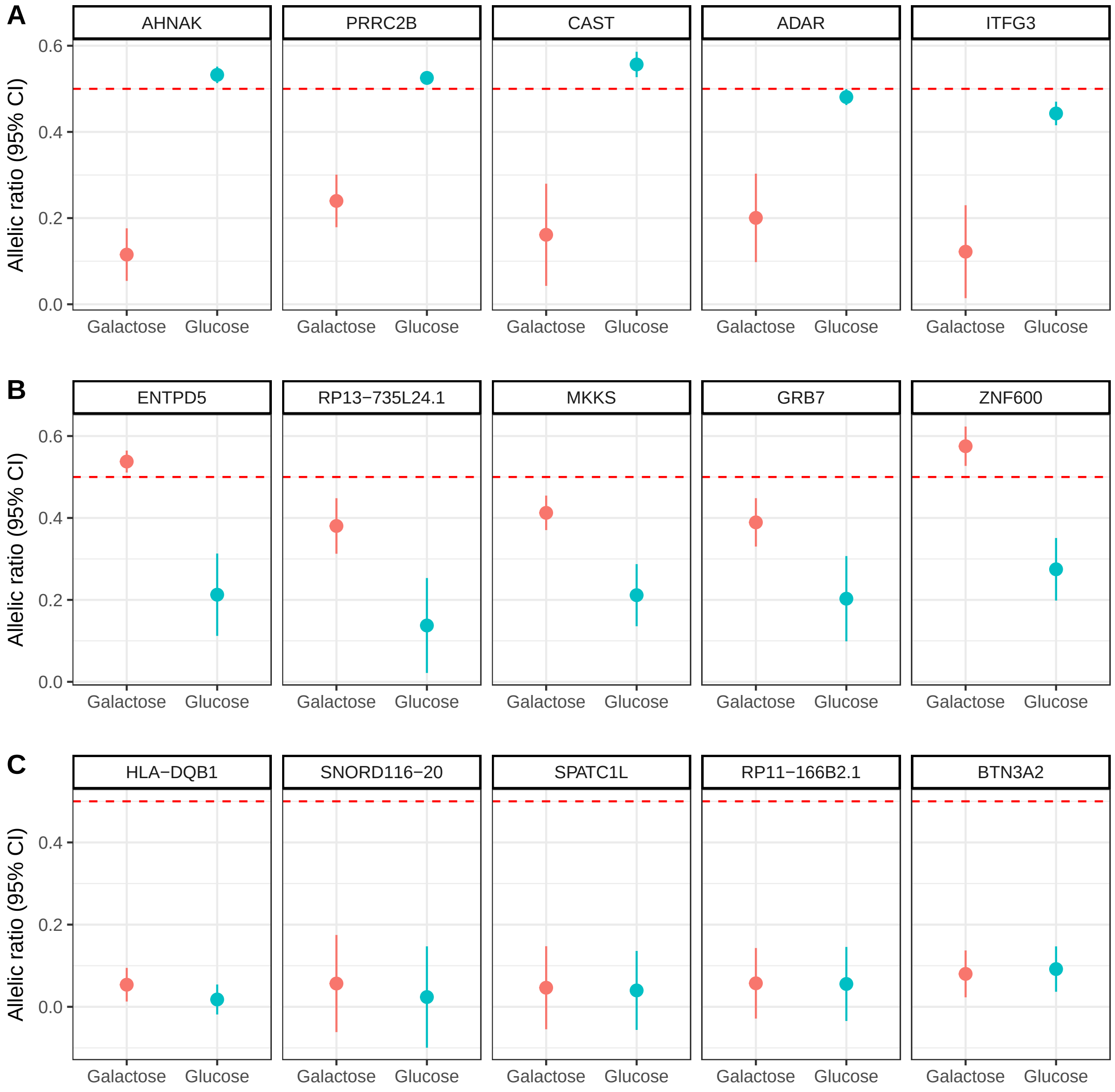

Supplementary Figure 5: Examples of response eQTL. We plotted the allelic ratios for the top five hits for galactose-specific, glucose-specific, and shared eQTL. The treatment-specific eQTL are ranked by difference in allelic imbalances across two conditions, and the shared eQTL are ranked by the sum in allelic imbalances.

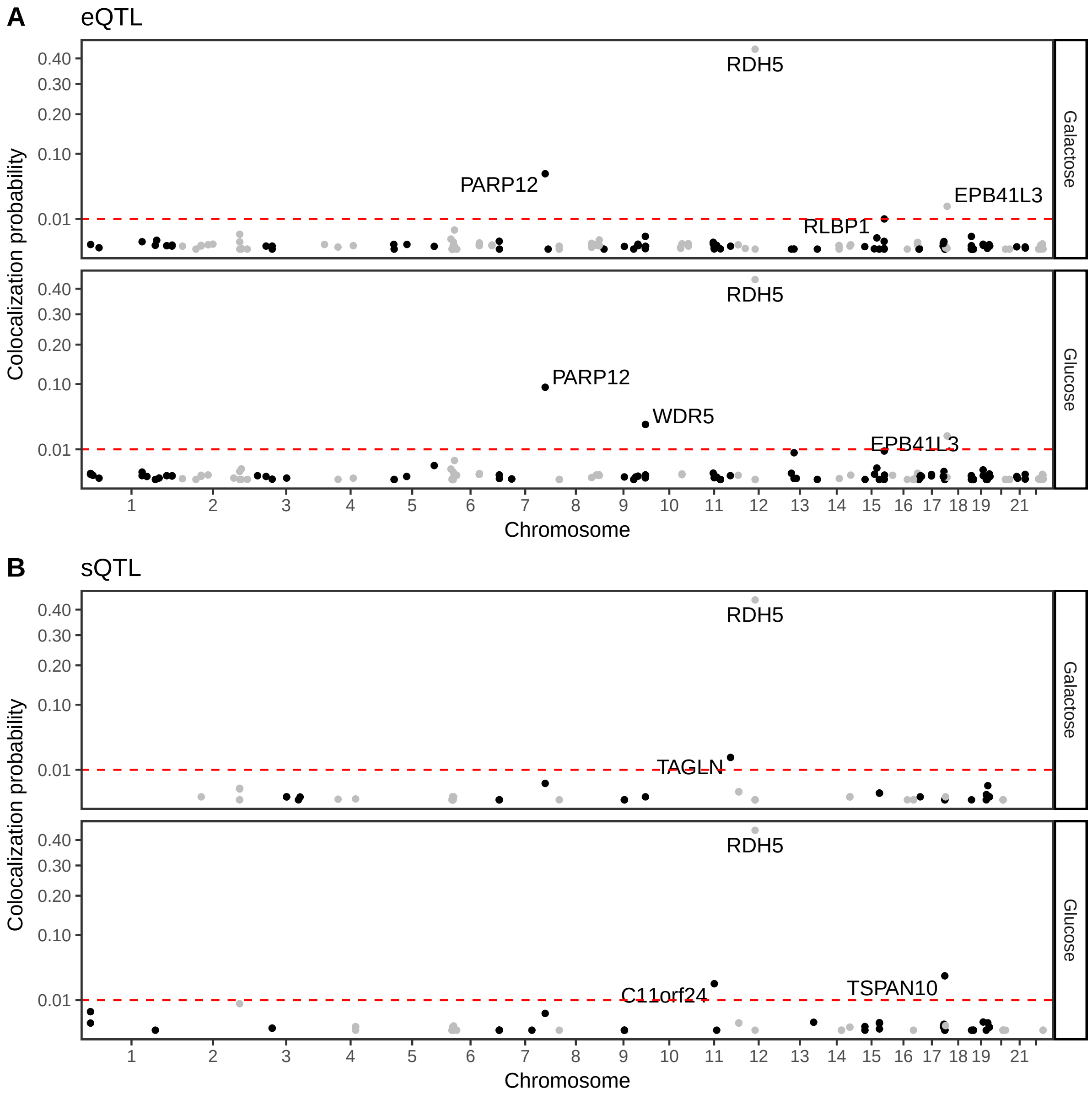

Supplementary Figure 6: Manhattan plots of eCAVIAR colocalization probability for AMD GWAS. (a) eQTL colocalizations. **(b)** sQTL colocalizations.

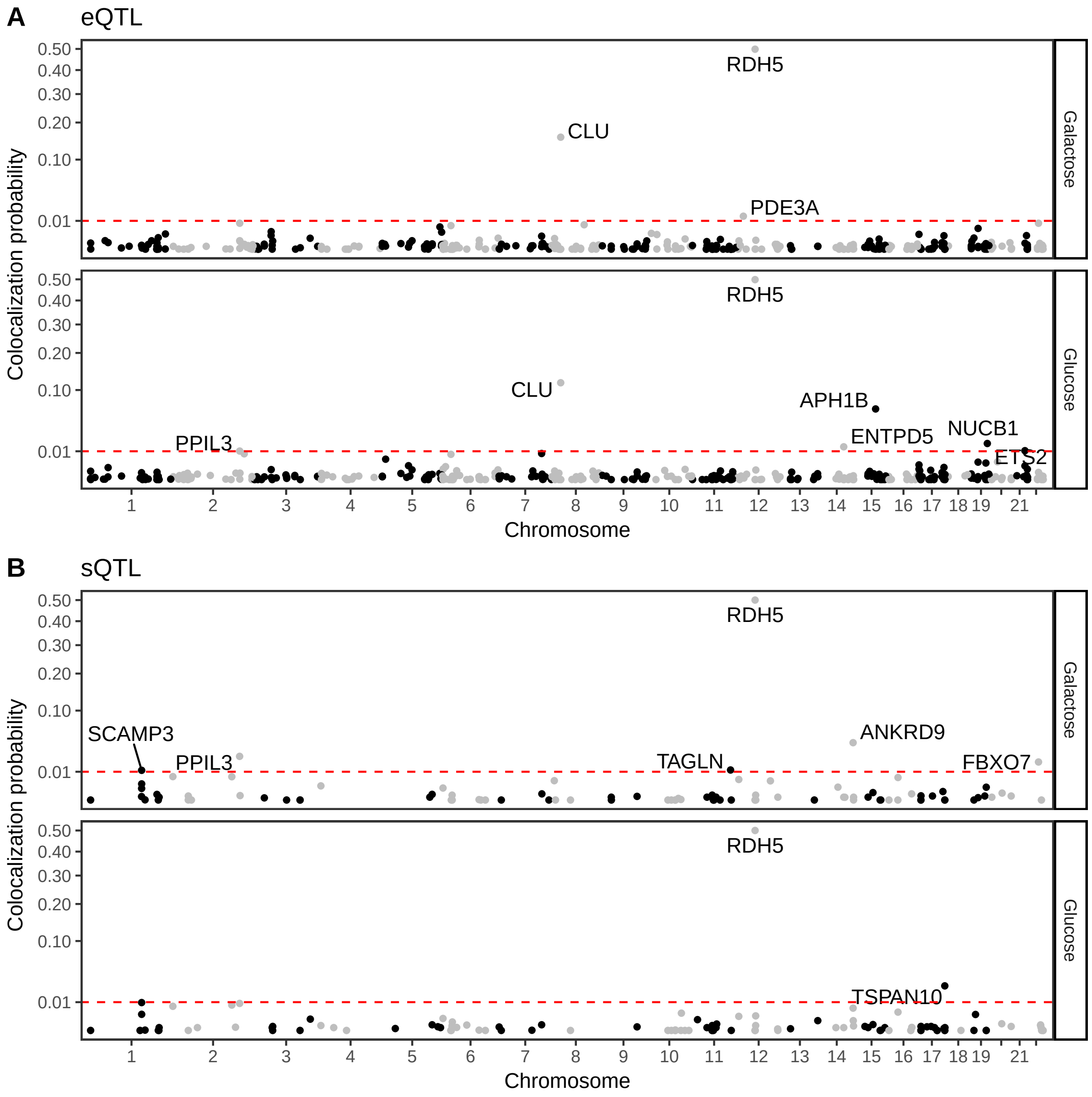

Supplementary Figure 7: Manhattan plots of eCAVIAR colocalization probability for myopia GWAS. **(a)** eQTL colocalizations. **(b)** sQTL colocalizations.

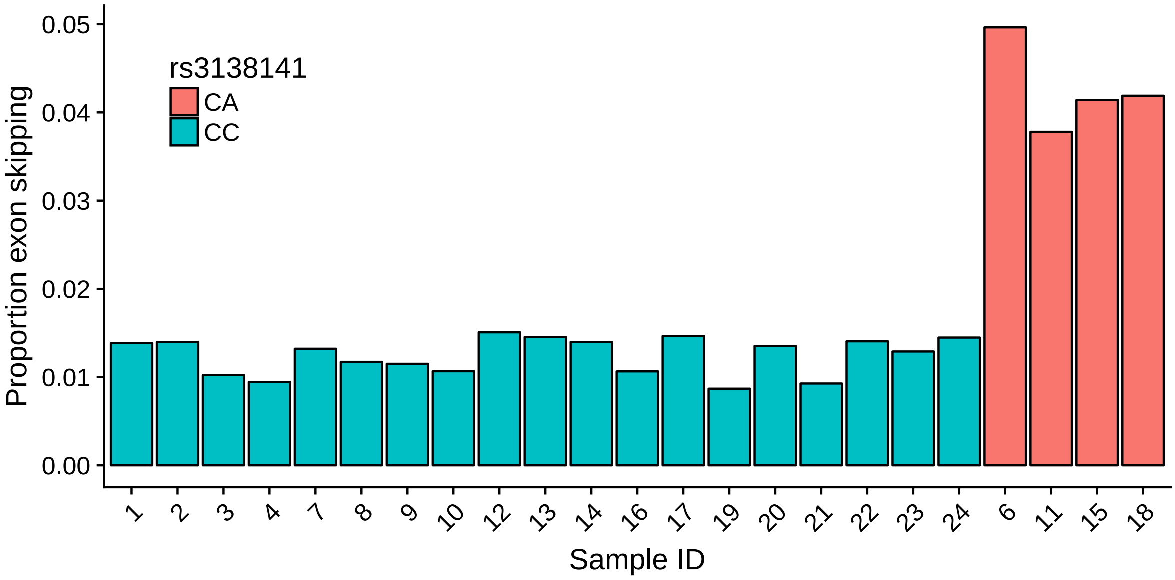

Supplementary Figure 8: *RDH5* exon 3 splicing levels stratified by rs3138141 genotype.

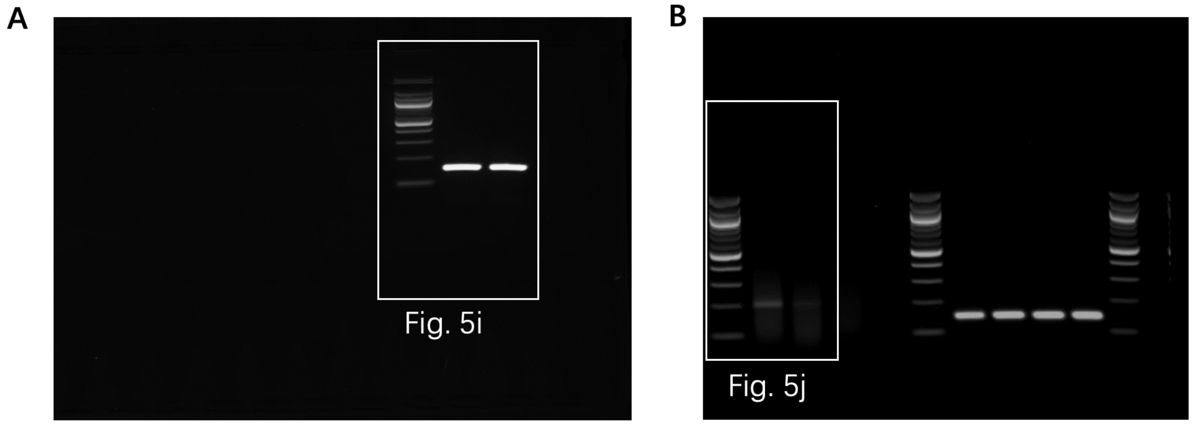

Supplementary Figure 9: (a) Original uncropped gel image showing *RHD5* normal isoform amplified from CHX or DMSO treated ARPE-19 cells. (b) Original uncropped gel image showing *RHD5* mis-spliced isoform amplified from CHX or DMSO treated ARPE-19 cells.

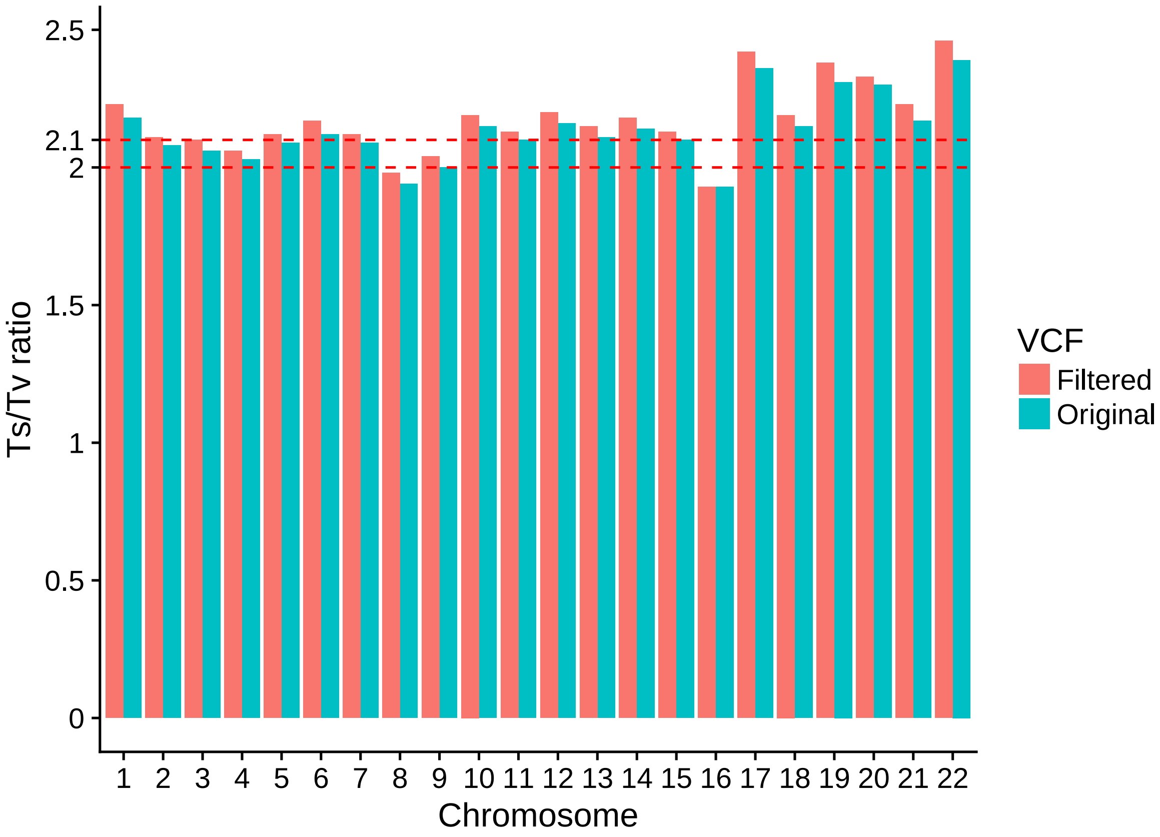

Supplementary Figure 10: Ts/Tv ratio for imputed VCF files. We performed calculated Ts/Tv ratio for the imputed VCF file prior to and after filtering. All but chromosome 8 and 16 in the post-filter VCF files have Ts/Tv ratio greater than 2.0. The ideal Ts/Tv ratio for whole-genome genotyping ranges from 2.0 - 2.1, and higher ratios indicate that the datasets has more known variants.

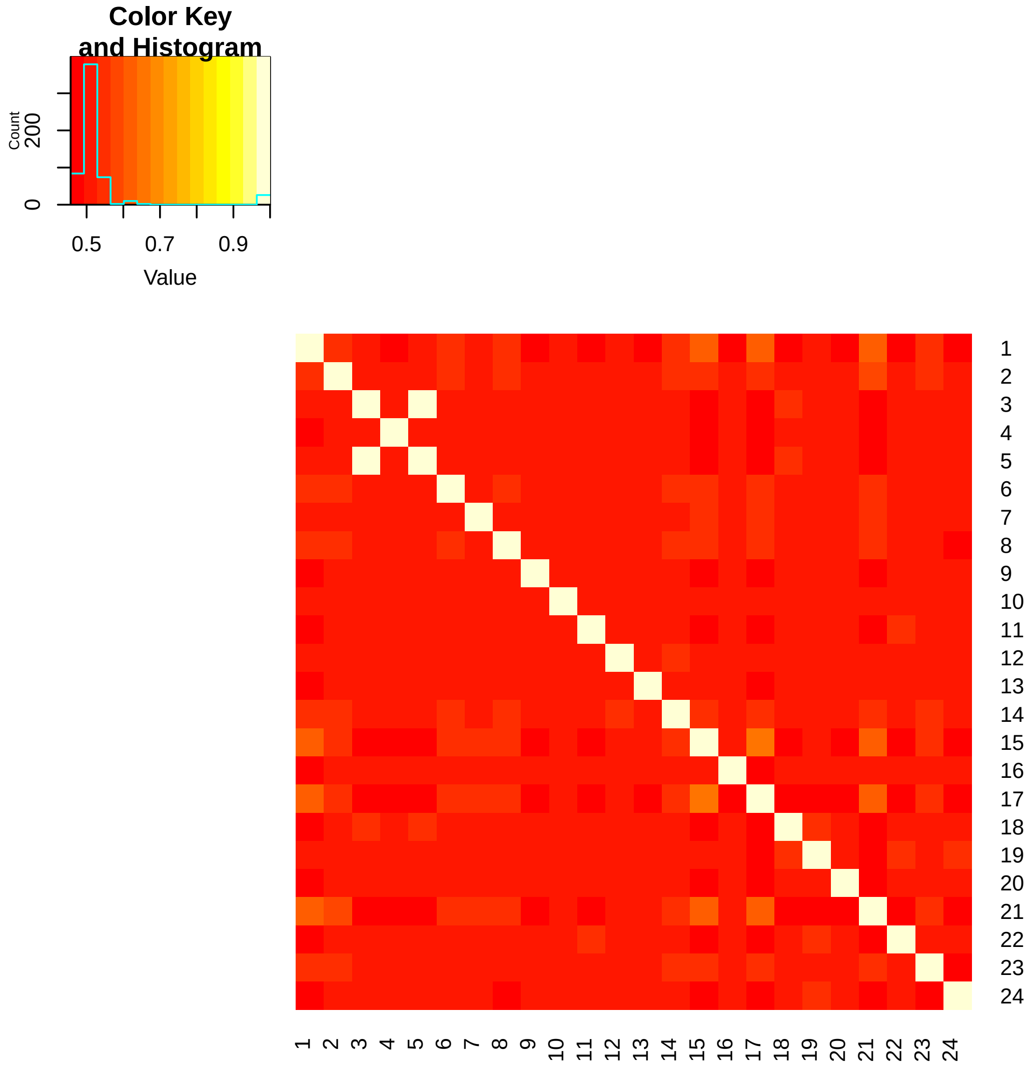

Supplementary Figure 11: Genotype correlation across 24 RPE samples. We determined sample duplications using the correlation of genotypes. Two samples were duplicates of each other and one was removed arbitrarily.

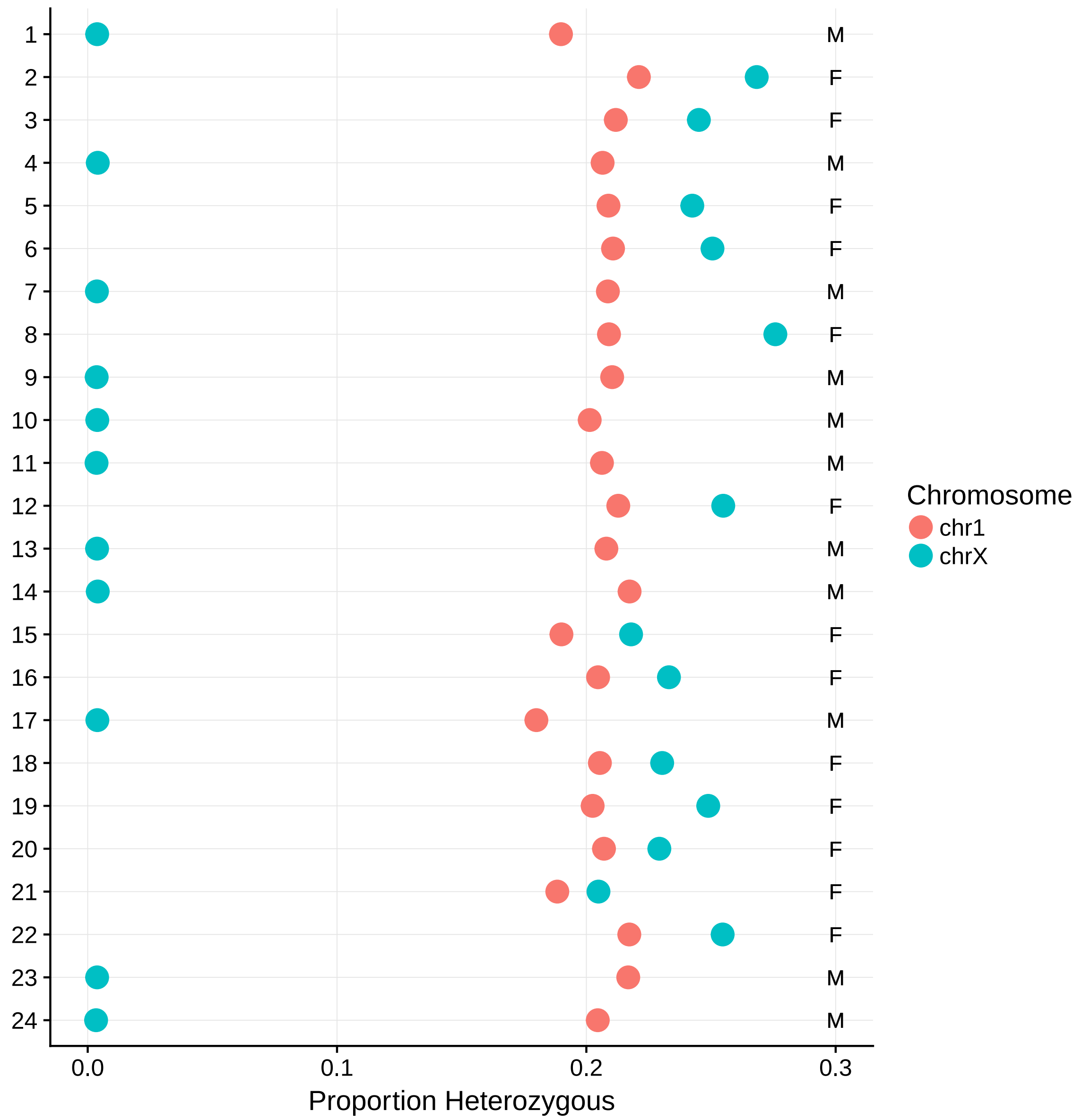

Supplementary Figure 12: Proportion of heterozygous SNPs in chromosome 1 and X. We determined the proportion of heterozygous SNPs on chromosome 1 and X, and defined male individuals as those with low heterozygosity (≈ 0) on chromosome X. Chromosome 1 was used to establish a baseline of heterozygosity.

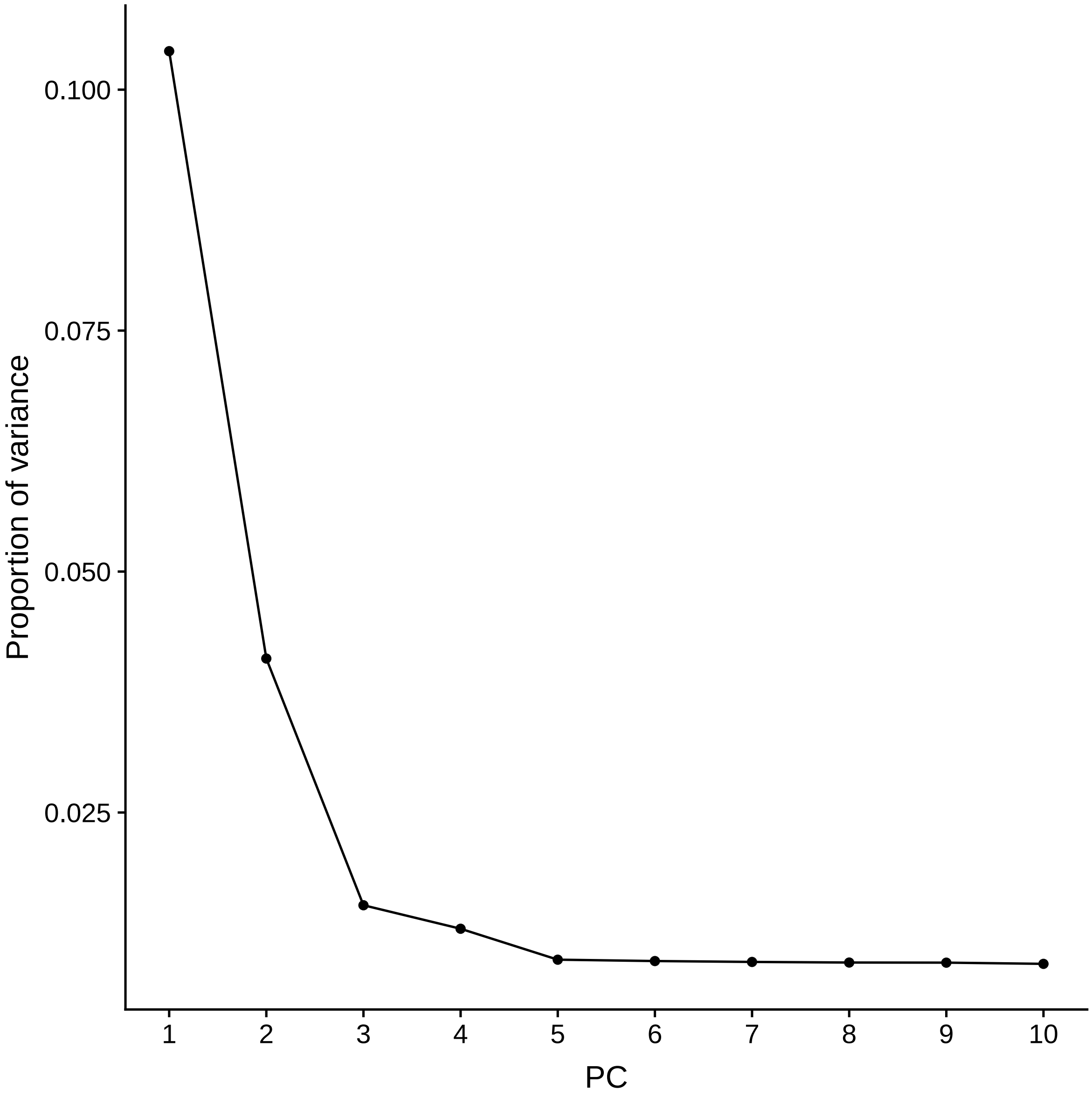

Supplementary Figure 13: Genotype principal components scree plot. The "elbow" appears at the third PC. Therefore, we used the top three PCs for downstream analysis.

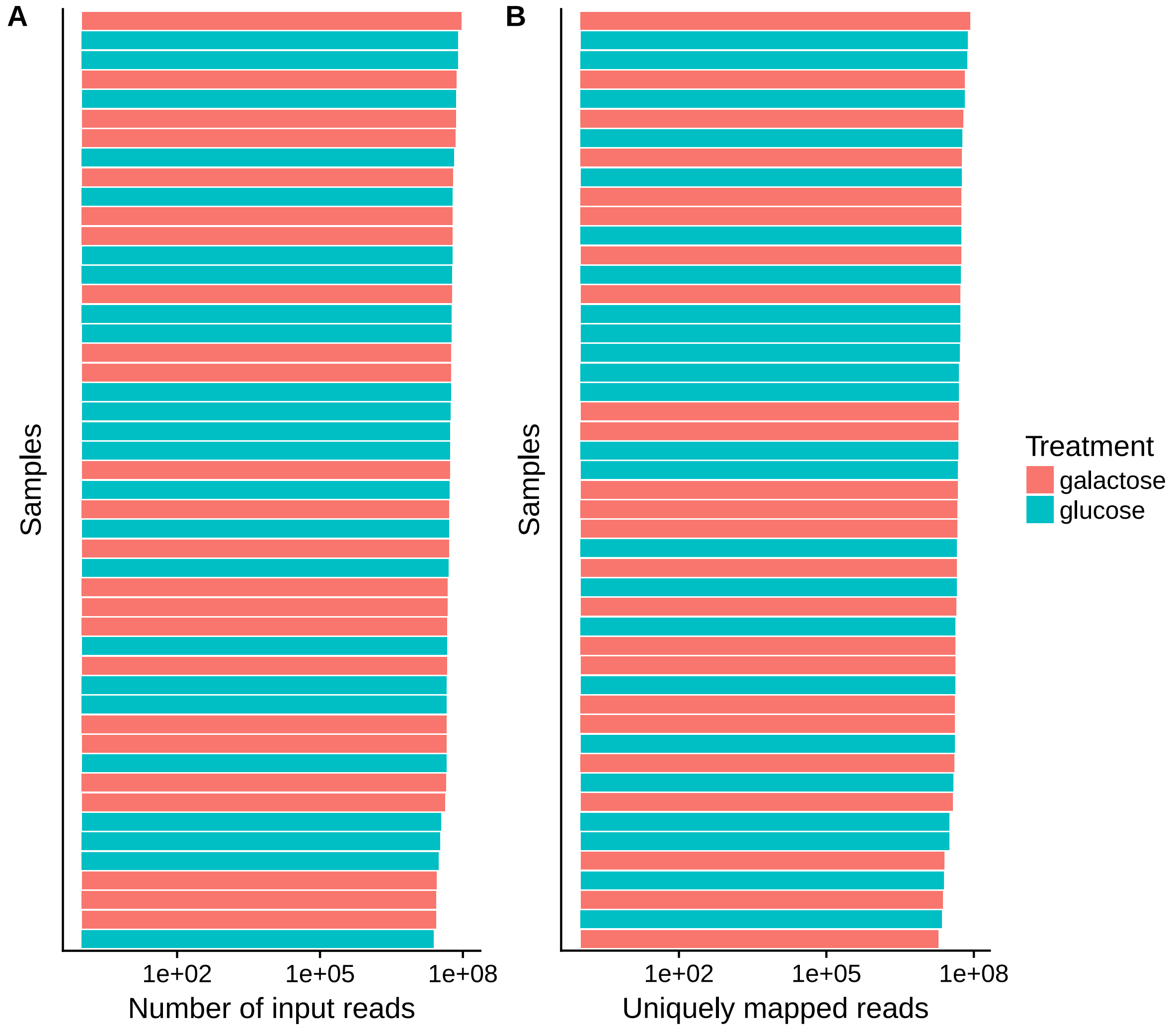

Supplementary Figure 14: RNA sequencing depth by sample. (a) We performed 75 bp paired-end sequencing on 48 RPE RNAseq libraries (24 samples for each glucose and galactose condition) to a median depth of 52.7 million reads (interquartile range: 45.5 to 60.1 million reads), for a total of 2.5 billion reads. **(b)** After alignment, a median of 46.8 millions (88.8%) reads were uniquely mapped, with a interquartile range of 41.0 to 55.2 million reads

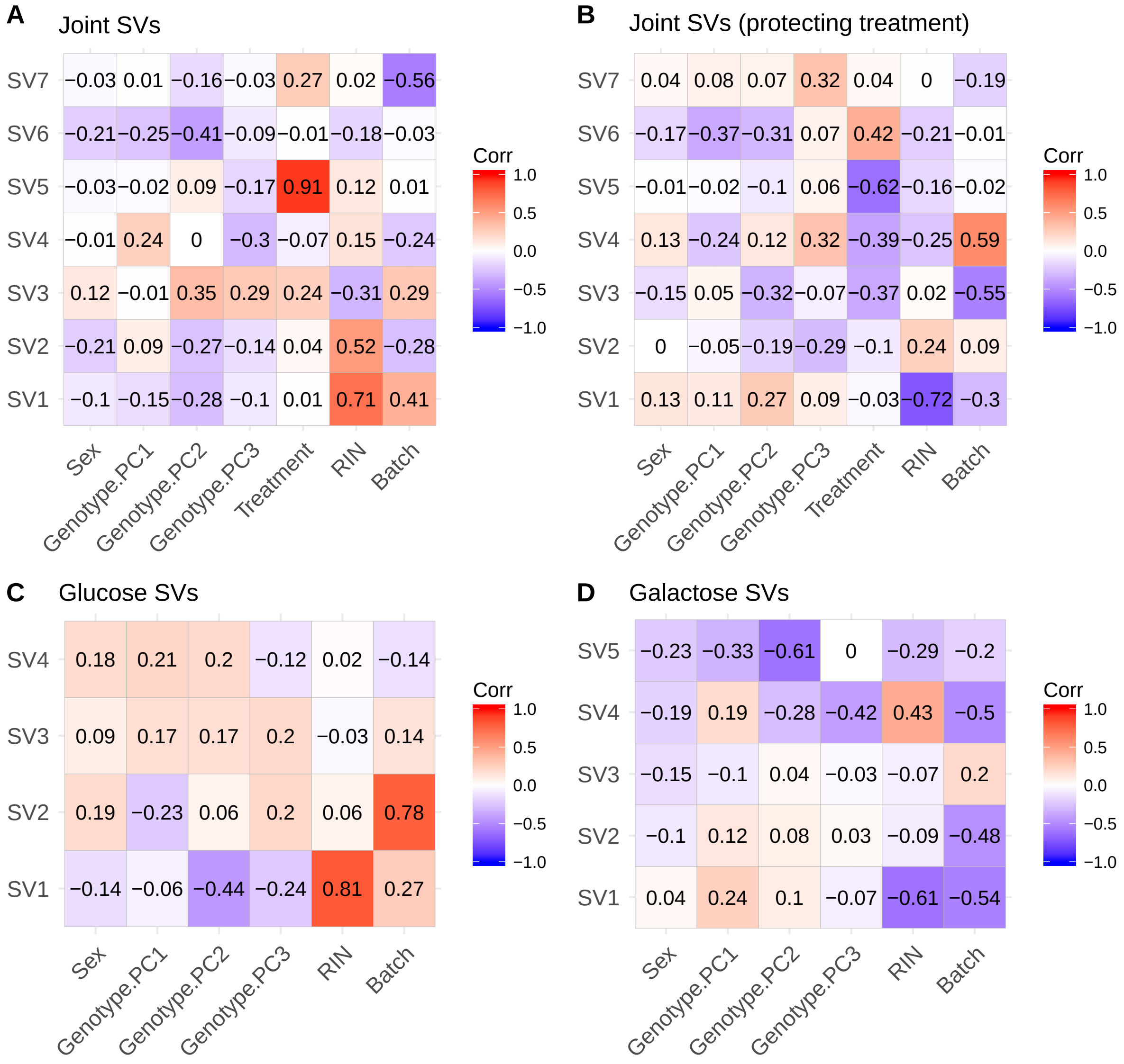

Supplementary Figure 15: Correlation between known and hidden factors. **(a)** Jointly inferred factors (seven in total) captures treatment (factor 5, $r = 0.91$), RIN (factor 1, $r = 0.71$) and batch effect (factor 7, $r = -0.56$), but does not capture sex or genotype PCs. **(b)** Jointly inferred factors protecting for treatment. Factor 5 remains correlated with treatment ($r = -0.62$), likely due to the broad and strong effect exerted by metabolic perturbation. **(c)** The glucose surrogate variables captured RIN (factor 1, $r = 0.81$) and batch (factor 2, $r = 0.78$), but not sex or the genotype PCs. **(d)** The galactose surrogate variables captured RIN (factor 1, $r = -0.61$) and batch (factor 1 and 4, $r = -0.54$ and -0.5, respectively), but not sex or ancestry.

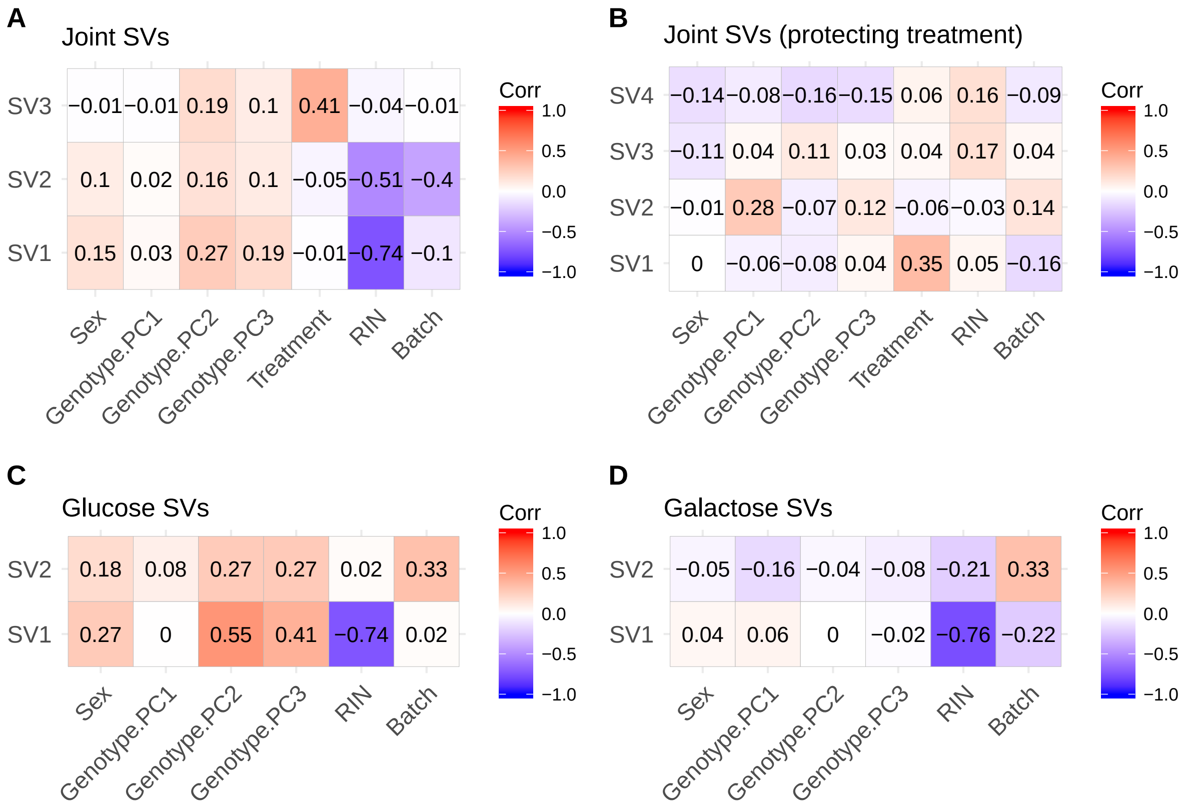

Supplementary Figure 16: Correlation between splicing surrogate variables and known covariates. **(a)** Correlation between SVs from the joint analysis and known covariates. SV1 correlated with RIN, and SV2 did not strongly correlate with known variables. **(b)** Correlation between SVs from the joint analysis (protecting the treatment) and known covariates. **(c)** Correlation between glucose SVs and known covariates. SV1 correlated with RIN, and SV2 did not strongly correlate with any known covariate. **(d)** Correlation between galactose SV and known covariates. SV1 correlated with RIN.

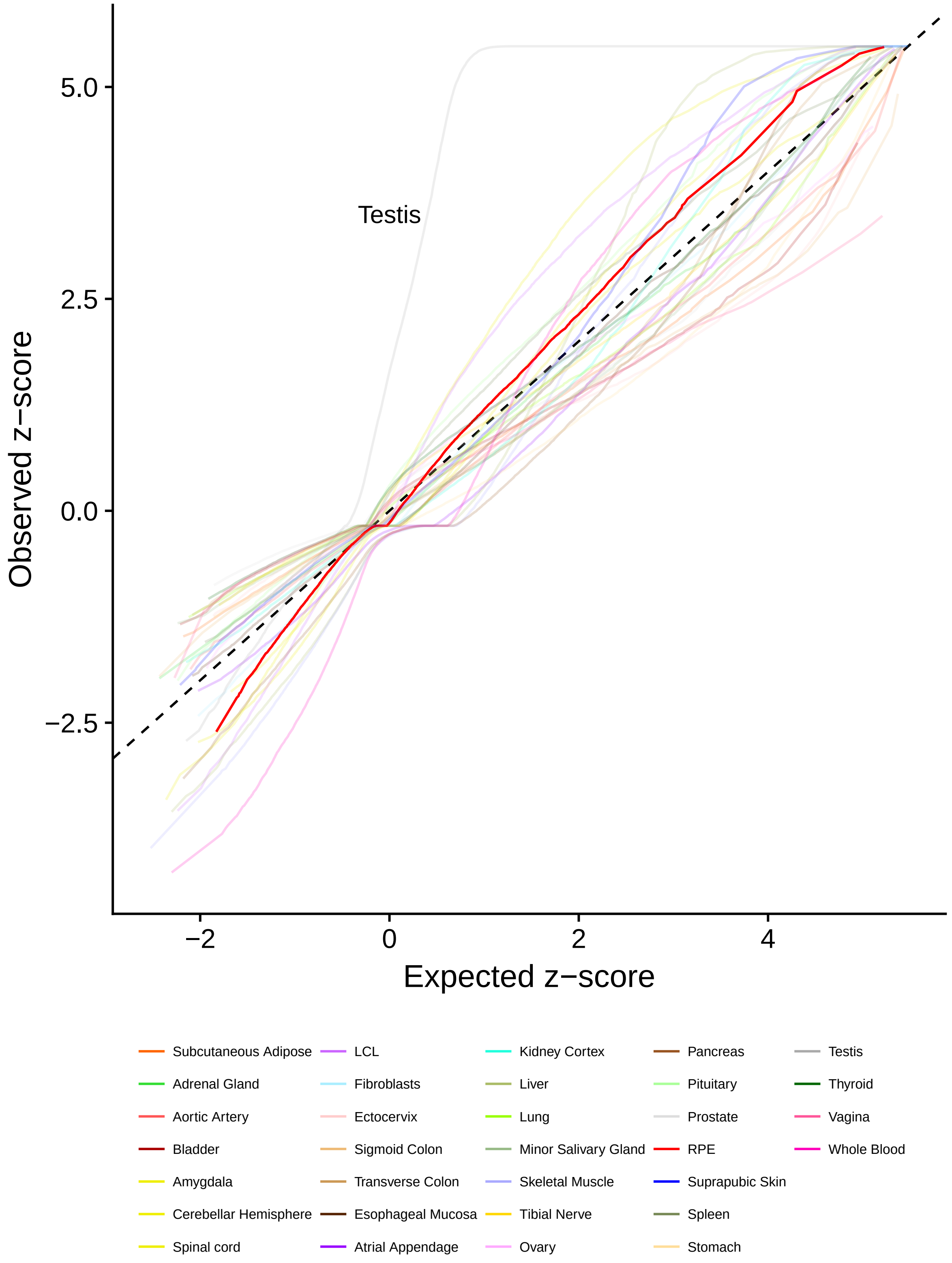

Supplementary Figure 17: Z-score QQ-plot. We visualized the z-score of each tissue against the average z-score across tissues (section ??). RPE z-score locate within the midst of the z-score from GTEx tissues. On the other hand, testis separate from other tissues as an outlier, as we expected.

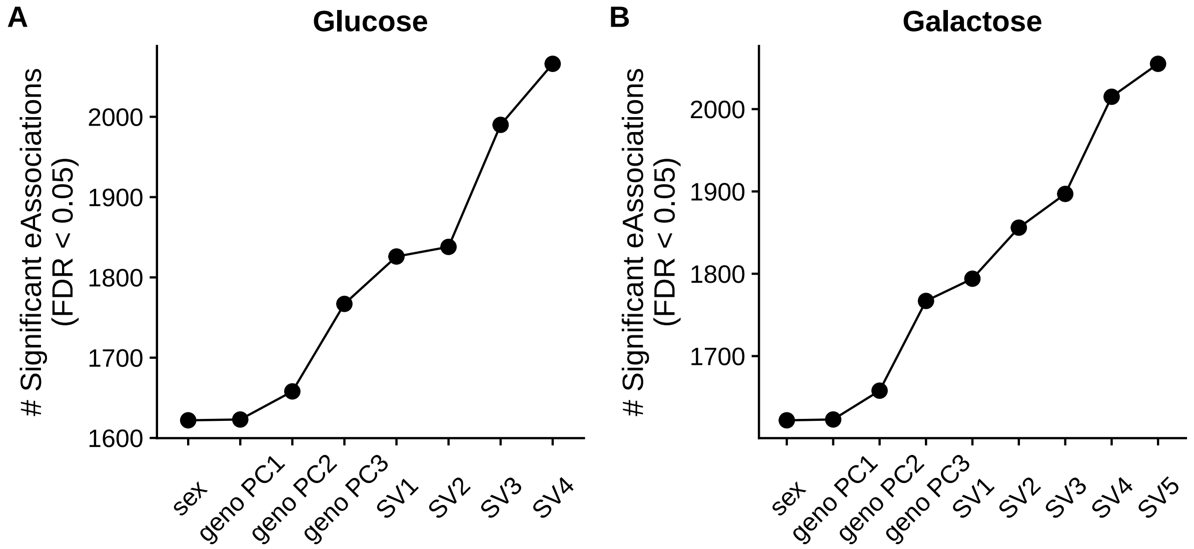

Supplementary Figure 18: Covariate selection for eQTL mapping. We selected covariates (sex, ancestry and hidden confounders) by empirically maximizing the power to detect eQTL. The number of eAssociations (defined as a SNP-gene pair that passed hierarchical multiple hypothesis testing by TreeQTL) increased monotonically as the number of covariates. Abbreviations: geno PC = genotype principal components; SV = surrogate variables.

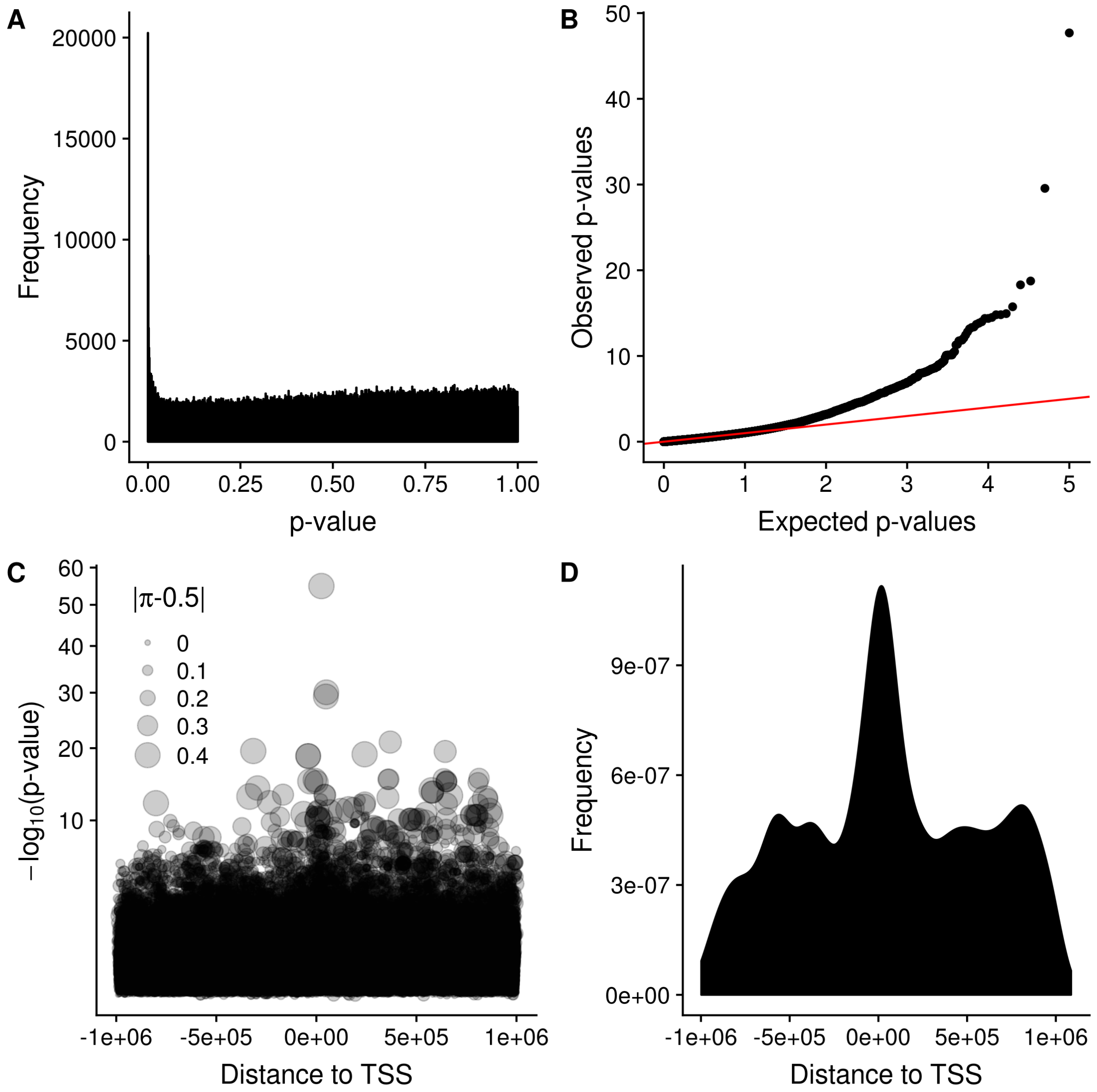

Supplementary Figure 19: eQTL quality control. **(a)** The eQTL p-value distribution shows slight conservativeness. **(b)** A QQ plot showing enrichment of low p-values compared with a uniform distribution. **(c)** eQTL with low p-values are enriched towards the TSS. **(D)** Significant eQTLs (defined here as p-value $< 1 \times 10^{-4}$ is enriched towards the TSS.

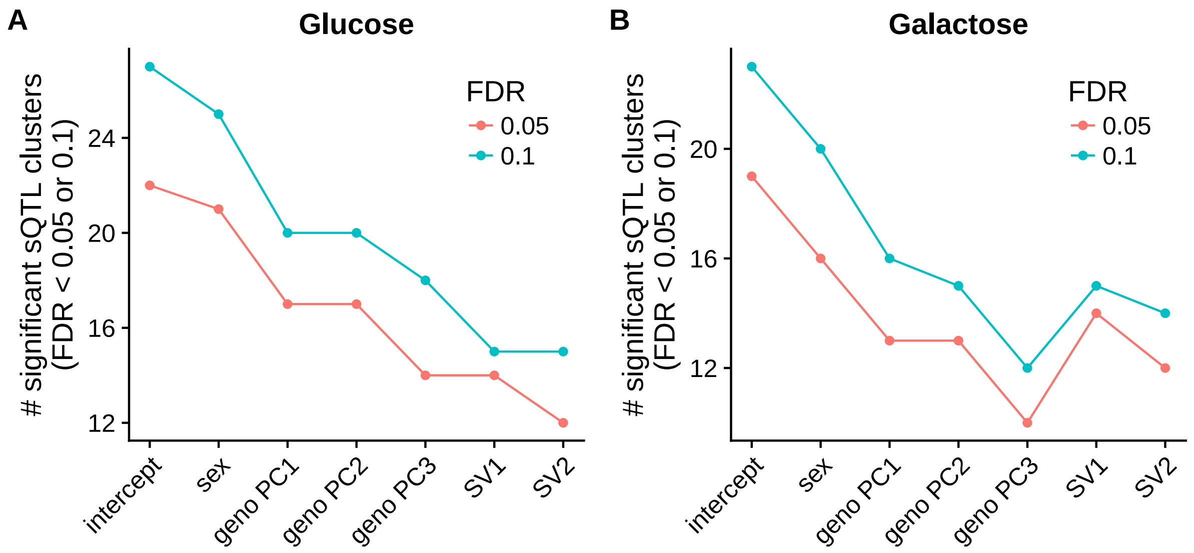

Supplementary Figure 20: sQTL covariate selection. The number of significant sQTL cluster (FDR < 0.05 and 0.01) decreased as the number of covariate increased for both (A) glucose and (B) galactose conditions.

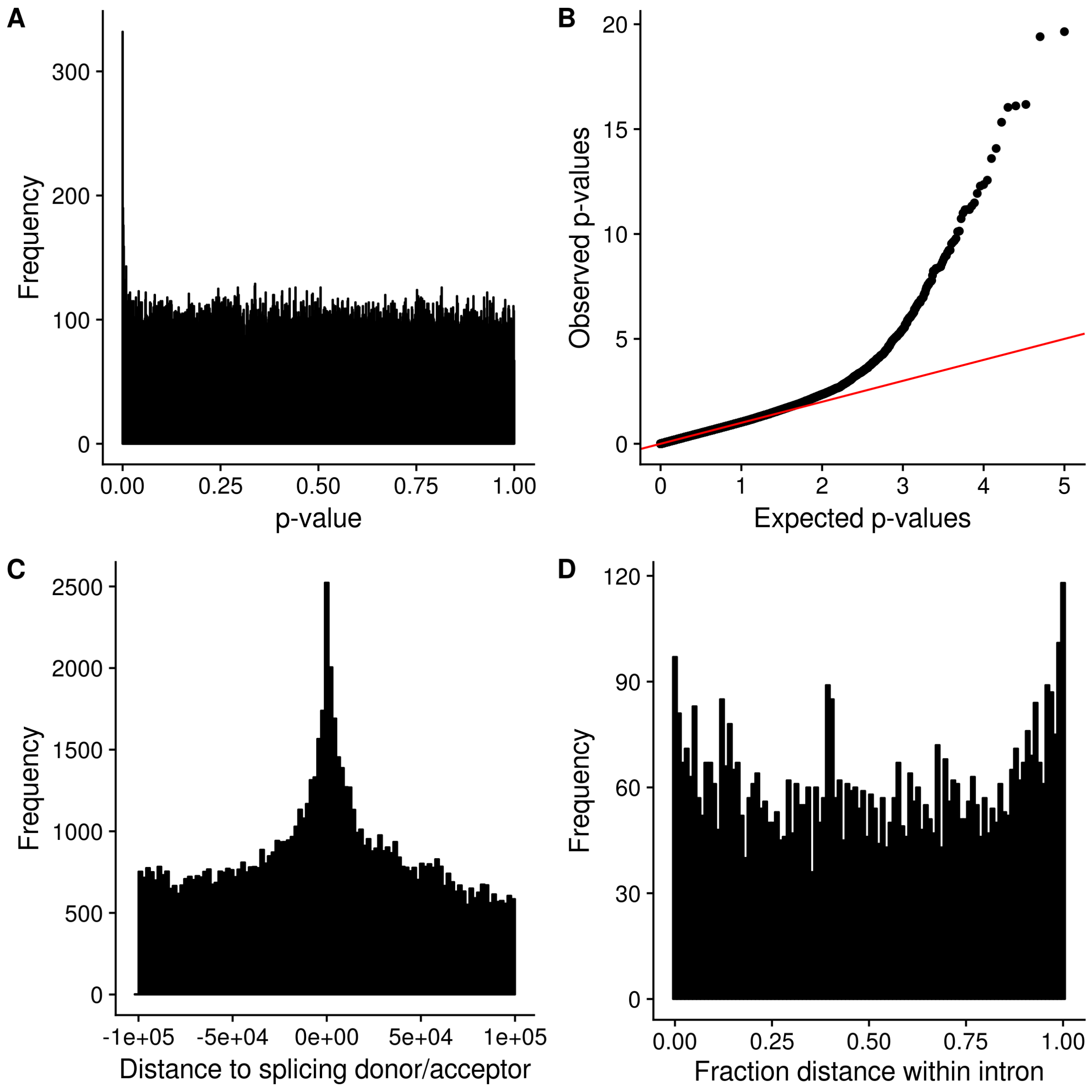

Supplementary Figure 21: sQTL quality control. **(a)** The sQTL p-value distribution shows uniform distribution and a spike at 0. **(b)** A QQ plot showing enrichment of low p-values compared with a uniform distribution. **(c)** sQTL with low p-values ($p - value < 10^{-4}$) are enriched towards the splicing donor and acceptor sites. **(d)** Intronic sQTL ($p - value < 10^{-4}$) are enriched around splicing donor and acceptor sites.

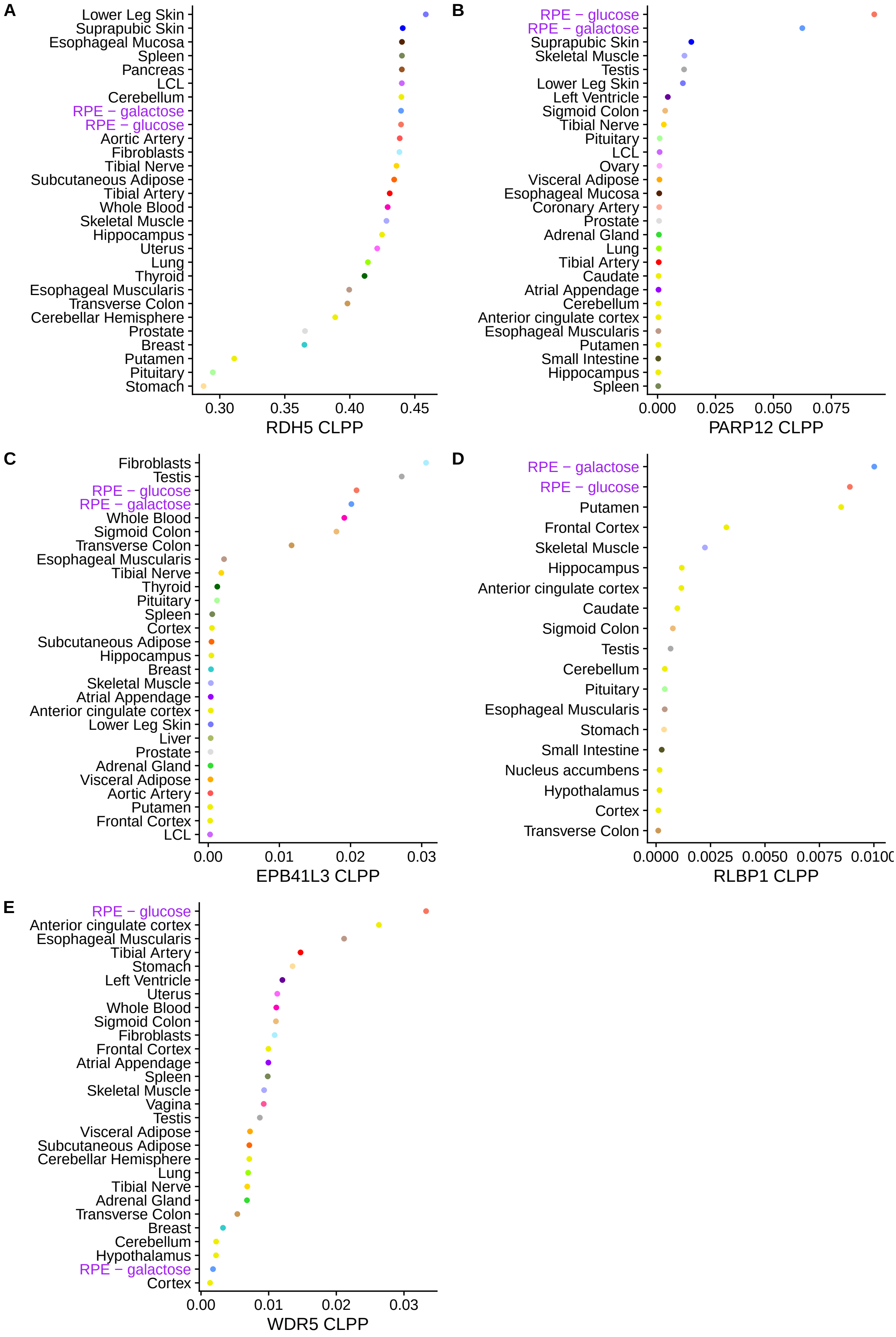

Supplementary Figure 22: Comparison of AMD colocalization posterior probability between GTEx and RPE.

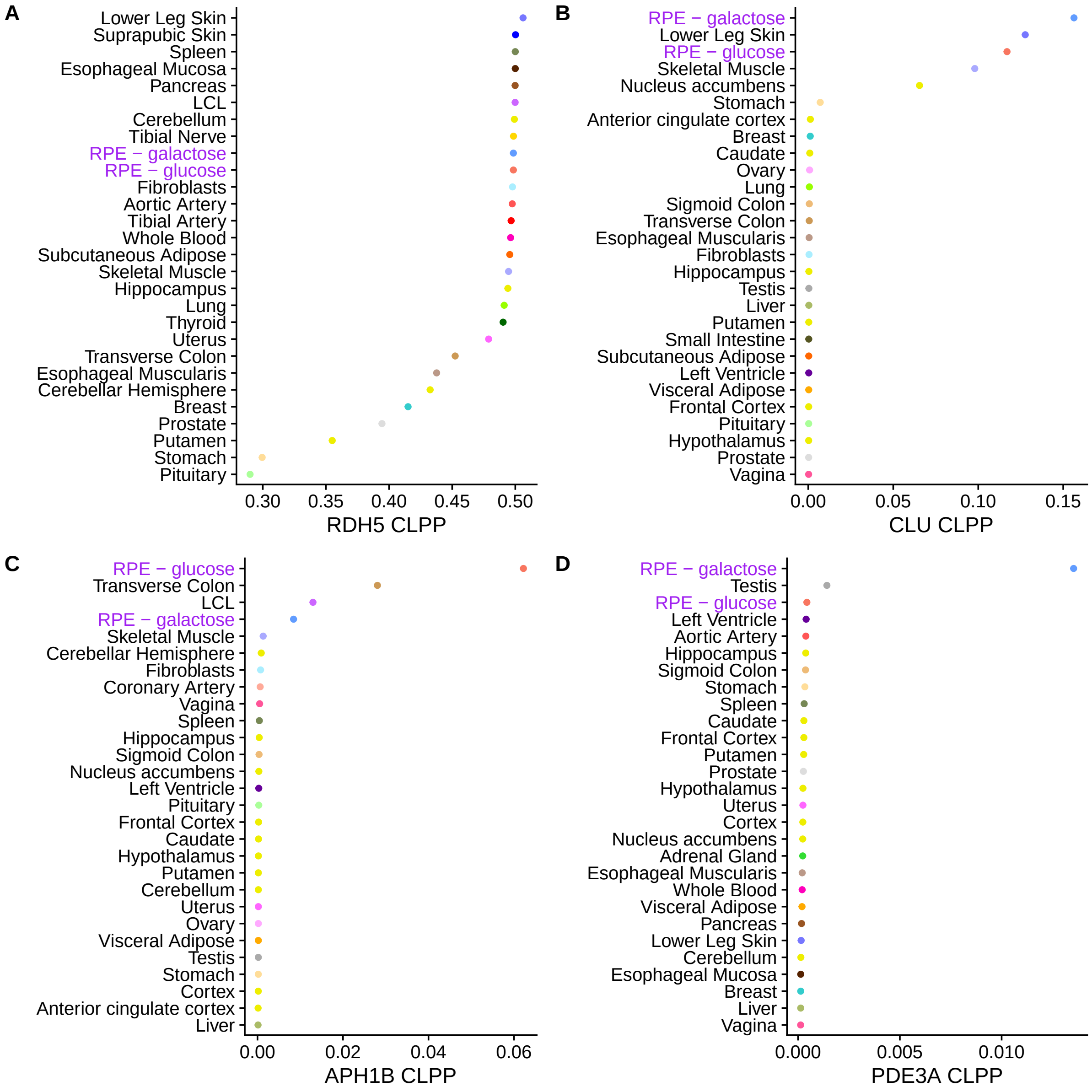

Supplementary Figure 23: Comparison of Myopia colocalization posterior probability between GTEx and RPE.

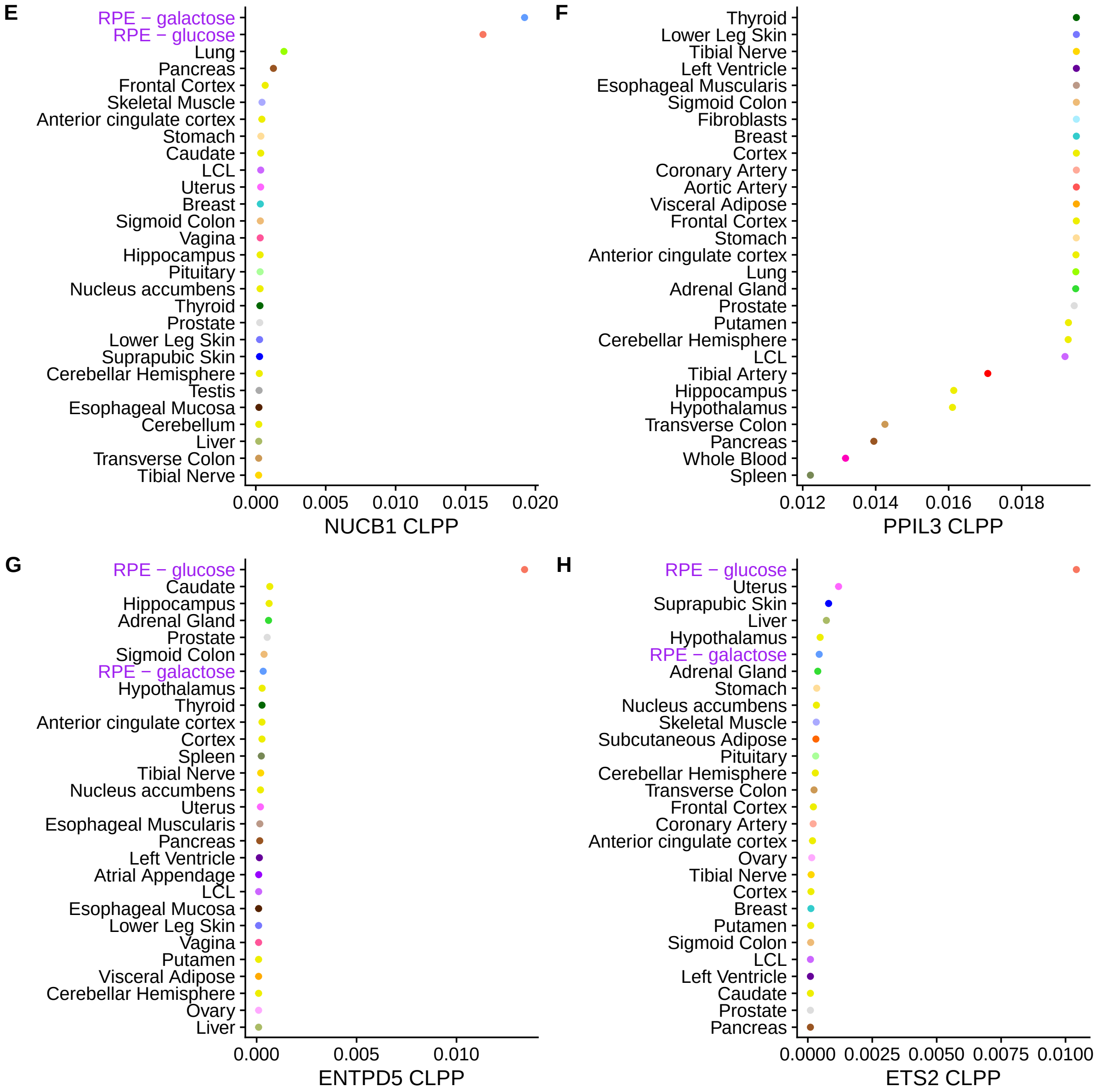

Supplementary Figure 23: (Continued) Comparison of Myopia colocalization posterior probability between GTEx and RPE. For (F), RPE - glucose and RPE - galactose have CLPP scores of 0.01 and 0.008, respectively. Their scores are less than the lowest score shown (Spleen) and therefore hidden.
