# Supplementary material for: Genetic analyses of human fetal retinal pigment epithelium gene expression suggest ocular disease mechanisms
Source: Commun Biol. 2019 May 20;2:186. doi: 10.1038/s42003-019-0430-6 (PMC6527609; doi:10.1038/s42003-019-0430-6)
Supplement: Supplementary file 2 — Description of Additional Supplementary Files [file 42003_2019_430_MOESM2_ESM.docx]

Supplementary Data 1: RPE cell lines

Supplementary Data 2: RNAseq libraries

Supplementary Data 3: RPE-selective genes

Supplementary Data 4: RPE-specific GO terms

Supplementary Data 5: Differential expression (Figs 2a and c)

Supplementary Data 6: Glucose eQTL (Figs 5b and e)

Supplementary Data 7: Galactose eQTL

Supplementary Data 8: Motif enrichment

Supplementary Data 9: Glucose sQTL (Figs 5c and f)

Supplementary Data 10: Galactose sQTL

Supplementary Data 11: AMD colocalization (Fig. 5a)

Supplementary Data 12: Myopia colocalization (Fig. 5d)

Supplementary Data 13: Fig. 1a data

Supplementary Data 14: Fig. 1b data

Supplementary Data 15: Fig. 1c data

Supplementary Data 16: Fig. 1d data

Supplementary Data 17: Fig. 2b data

Supplementary Data 18: Fig. 3a data

Supplementary Data 19: Fig. 3b data

Supplementary Data 20: Fig. 3c data

Supplementary Data 21: Fig. 4a data

Supplementary Data 22: Fig. 4b data

Supplementary Data 23: Fig. 4c data

Supplementary Data 24: Fig. 4d data

Supplementary Data 25: Fig. 4e data

Supplementary Data 26: Fig. 4f data

Supplementary Data 27: Fig. 4g data

Supplementary Data 28: Fig. 5g data

Supplementary Data 29: Fig. 5h data

Supplementary Data 30: Fig. 5k data
