# Supplementary material for: Genetic analyses of human fetal retinal pigment epithelium gene expression suggest ocular disease mechanisms
Source: Commun Biol. 2019 May 20;2:186. doi: 10.1038/s42003-019-0430-6 (PMC6527609; doi:10.1038/s42003-019-0430-6)
Supplement: Supplementary file 5 — Reporting Summary [file 42003_2019_430_MOESM5_ESM.pdf]

nature research

Corresponding author(s): Douglas Vollrath and Stephen Montgomery

Last updated by author(s): Apr 10, 2019

Reporting Summary

Nature Research wishes to improve the reproducibility of the work that we publish. This form provides structure for consistency and transparency in reporting. For further information on Nature Research policies, see Authors & Referees and the Editorial Policy Checklist.

Statistics

For all statistical analyses, confirm that the following items are present in the figure legend, table legend, main text, or Methods section.

n/a	Confirmed	
☐	☒	The exact sample size (*n*) for each experimental group/condition, given as a discrete number and unit of measurement
☐	☒	A statement on whether measurements were taken from distinct samples or whether the same sample was measured repeatedly
☐	☒	The statistical test(s) used AND whether they are one- or two-sided<br>*Only common tests should be described solely by name; describe more complex techniques in the Methods section.*
☐	☒	A description of all covariates tested
☐	☒	A description of any assumptions or corrections, such as tests of normality and adjustment for multiple comparisons
☐	☒	A full description of the statistical parameters including central tendency (e.g. means) or other basic estimates (e.g. regression coefficient) AND variation (e.g. standard deviation) or associated estimates of uncertainty (e.g. confidence intervals)
☐	☒	For null hypothesis testing, the test statistic (e.g. *F*, *t*, *r*) with confidence intervals, effect sizes, degrees of freedom and *P* value noted<br>*Give P values as exact values whenever suitable.*
☒	☐	For Bayesian analysis, information on the choice of priors and Markov chain Monte Carlo settings
☒	☐	For hierarchical and complex designs, identification of the appropriate level for tests and full reporting of outcomes
☐	☒	Estimates of effect sizes (e.g. Cohen's *d*, Pearson's *r*), indicating how they were calculated

Our web collection on statistics for biologists contains articles on many of the points above.

Software and code

Policy information about availability of computer code

Data collection	Code to reproduce all analyses in this manuscript has been deposited on GitHub: https://github.com/boxiangliu/rpe
Data analysis	Code to reproduce all analyses in this manuscript has been deposited on GitHub: https://github.com/boxiangliu/rpe

For manuscripts utilizing custom algorithms or software that are central to the research but not yet described in published literature, software must be made available to editors/reviewers. We strongly encourage code deposition in a community repository (e.g. GitHub). See the Nature Research guidelines for submitting code & software for further information.

Data

Policy information about availability of data

All manuscripts must include a data availability statement. This statement should provide the following information, where applicable:

- Accession codes, unique identifiers, or web links for publicly available datasets
- A list of figures that have associated raw data
- A description of any restrictions on data availability

All relevant data are available in the Supplementary Data files. Full eQTL and sQTL summary statistics have been deposited into Box: https://stanford.box.com/s/asrxy0o66xxe1j7mfj56p3z3d405gijj and are available at http://montgomerylab.stanford.edu/resources.html. RNAseq data can be downloaded via GEO accession number GSE129479.

Field-specific reporting

Please select the one below that is the best fit for your research. If you are not sure, read the appropriate sections before making your selection.

☒ Life sciences ☐ Behavioural & social sciences ☐ Ecological, evolutionary & environmental sciences

For a reference copy of the document with all sections, see nature.com/documents/nr-reporting-summary-flat.pdf

Life sciences study design

All studies must disclose on these points even when the disclosure is negative.

Sample size	RPE cells were sampled from 24 donors based on recommendation in van de Geijn (2015) Nature Methods.
Data exclusions	One unexpected duplicated sample was removed, reducing the number of samples to 23.
Replication	A substantial majority of the eQTLs we identified are present under two different metabolic conditions. We compared our eQTL findings with GTEx. All but 3 eQTLs were replicated in GTEx at FDR < 0.05. We believe that these 3 are valid eQTLs that are present in the RPE, but not in GTEx. Also, 72.5% of the shared RPE eGenes we identified are present in the EyeGEx dataset derived from neural retina (see text).
Randomization	We used a standard eQTL mapping pipeline established previously (Kumasaka, 2016, Nature Genetics) and controlled for ancestry, sex and age.
Blinding	The investigator who created and sequenced the RNA-seq libraries was aware of the treatment condition for each sample. This was essential to our study design because it enabled us to sequence samples from the same donor but different treatment conditions at the same time, thus avoiding confounding of differential gene expression analysis by sequencing batch effects. The donor genotypes were completely masked (unknown) to the investigator who generated the RNA-seq data. In fact, an unexpected duplicate line was only discovered after joint analysis of genotypes and RNA-seq data when all of the data collection for differential expression and e/sQTL analysis had been completed.

Reporting for specific materials, systems and methods

We require information from authors about some types of materials, experimental systems and methods used in many studies. Here, indicate whether each material, system or method listed is relevant to your study. If you are not sure if a list item applies to your research, read the appropriate section before selecting a response.

Materials & experimental systems

n/a	Involved in the study	
☒	☐	Antibodies
☐	☒	Eukaryotic cell lines
☒	☐	Palaeontology
☒	☐	Animals and other organisms
☒	☐	Human research participants
☒	☐	Clinical data

Methods

n/a	Involved in the study	
☒	☐	ChIP-seq
☒	☐	Flow cytometry
☒	☐	MRI-based neuroimaging

Eukaryotic cell lines

Policy information about cell lines

Cell line source(s)	ARPE-19 cells were obtained from ATCC (CRL-2302)
Authentication	The cells were obtained directly from ATCC within the past year. They exhibit the expected cobblestone morphology and slight pigmentation when differentiated by standard protocols.
Mycoplasma contamination	ARPE-19 cells were fixed, stained with DAPI and imaged by fluorescence microscopy. No evidence of mycoplasma contamination was seen.
Commonly misidentified lines (See ICLAC register)	ARPE-19 is not listed in Version 9 of the Register of Misidentified Cell Lines released October 14, 2018.
